# Biomimetic [MFe_3_S_4_]^3+^ Cubanes (M = V/Mo) as Catalysts for a Fischer–Tropsch-like Hydrocarbon Synthesis—A Computational Study

**DOI:** 10.1021/acs.inorgchem.4c04995

**Published:** 2024-12-27

**Authors:** Maxim Barchenko, Thomas Malcomson, Patrick J. O’Malley, Sam P. de Visser

**Affiliations:** † Department of Chemistry, School of Natural Sciences, The University of Manchester, Oxford Road, Manchester M13 9PL, U.K.; ‡ Manchester Institute of Biotechnology, The University of Manchester, 131 Princess Street, Manchester M1 7DN, U.K.; § Department of Chemical Engineering, The University of Manchester, Oxford Road, Manchester M13 9PL, U.K.

## Abstract

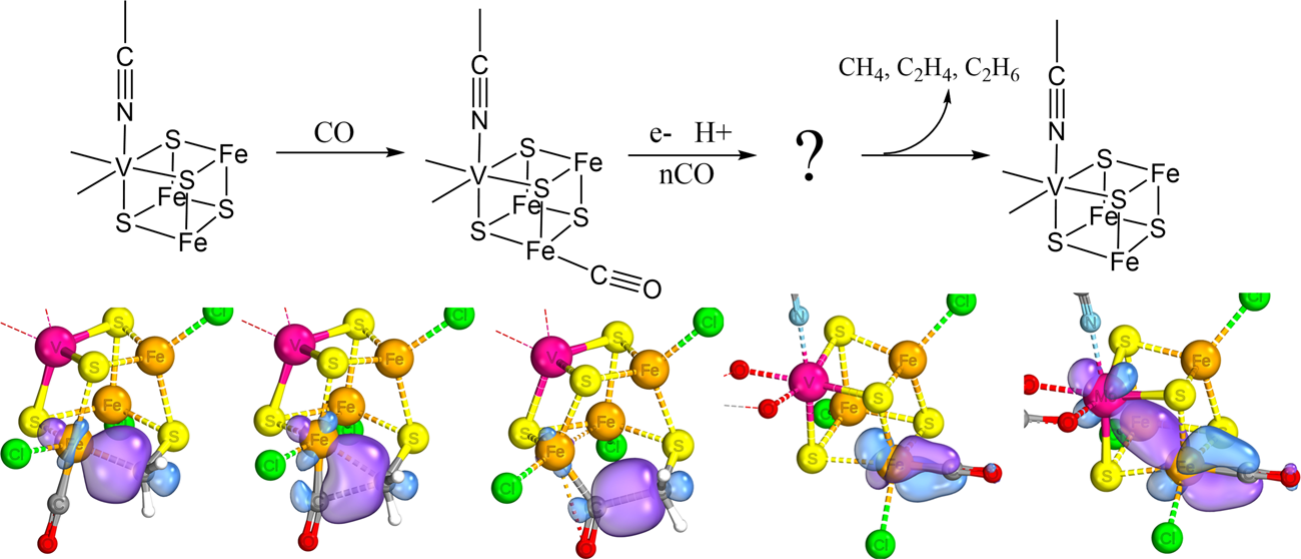

Nitrogenase is the enzyme primarily responsible for reducing atmospheric nitrogen to ammonia. There are three general forms of nitrogenase based on the metal ion present in the cofactor binding site, namely, molybdenum-dependent nitrogenases with the iron–molybdenum cofactor (FeMoco), the vanadium-dependent nitrogenases with FeVco, and the iron-only nitrogenases. It has been shown that the vanadium-dependent nitrogenases tend to have a lesser efficacy in reducing dinitrogen but a higher efficacy in binding and reducing carbon monoxide. In biomimetic chemistry, [MFe_3_S_4_] (M = Mo/V) cubanes have been synthesized, studied, and shown to be promising mimics of some of the geometric and electronic properties of the nitrogenase cofactors. In this work, a density functional theory (DFT) study is presented on Fischer–Tropsch catalysis by these cubane complexes by studying CO binding and reduction to hydrocarbons. Our work implies that molybdenum has stronger binding interactions with the iron–sulfur framework of the cubane, which results in easier reduction of substrates like N_2_H_4_. However, this inhibits the binding and activation of CO, and hence, the molybdenum-containing complexes are less suitable for Fischer–Tropsch catalysis than vanadium-containing complexes.

## Abstract 2

This work contains density functional theory calculations on Mo- and V-containing Fe_3_S_4_ single cubanes binding and activating CO, then subsequently catalyzing its reduction to CH_4_, C_2_H_4_, and C_2_H_6_. The calculations suggest the most favored stepwise mechanism for the transformation of CO when activated by these cubanes, with implications for similar iron−sulfur systems and the nitrogenase enzymes.

## 1 Introduction

The nitrogenase enzymes are responsible for the biological conversion of atmospheric dinitrogen (N_2_) to ammonia (NH_3_). There are three main types of nitrogenases found in nature—the molybdenum-dependent,^1^ the vanadium-dependent,^2^ and the iron-only,^3^ utilizing the respective metal ions in the Fe/S frameworks of their cofactors, such as the iron–molybdenum cofactor (FeMoco), which are believed to be where the binding and reduction of N_2_ takes place.^4^ Experimental evidence has shown that the Mo-dependent nitrogenases have the highest reactivity and turnover of all nitrogenases with regard to the fixing of atmospheric nitrogen.^5^ Furthermore, the V-dependent nitrogenases are typically found in Mo-deficient conditions, whereas the iron-only nitrogenases are mainly found in environments that lack both Mo and V.^6^ Owing to its earlier discovery and better efficiency, there have been significantly more studies on the Mo-dependent nitrogenase. Despite that, even for the Mo-dependent nitrogenase, many aspects of the mechanism by which it is able to bind and reduce dinitrogen remain uncertain.^7−11^

**Figure 1:**
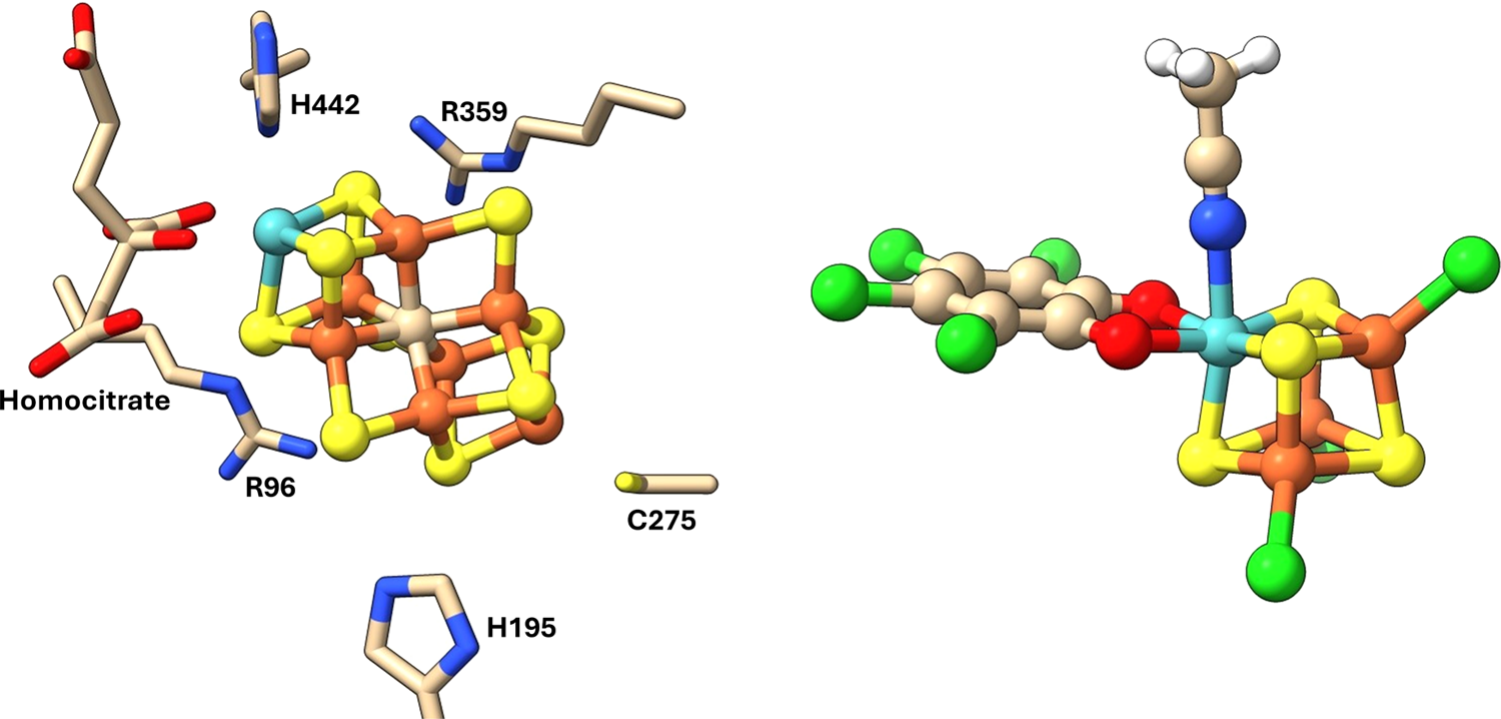
Depictions of the active site of FeMoco (left) and the [MFe_3_S_4_]^3+^ cubanes used as the model in the study with a bound acetonitrile ligand (right). Color scheme: turquoise = Mo/V, yellow = S, dark orange = Fe, green = Cl, red = O, gray = C, blue = N.

Biomimetic models are useful synthetic clusters that have the coordination environment of enzyme active sites but lack protein. These are commonly studied to gain insight into reaction mechanisms and spectroscopic properties of short-lived enzymatic intermediates.^12−21^ Early studies showed that [MoFe_3_S_4_]^3+^ cubanes possess certain properties in common with the nitrogenase cofactors, making them potentially useful as mimics for future study of nitrogenase function and development of synthetic catalysts.^22−24^ The [MoFe_3_S_4_]^3+^ complexes are experimentally known to catalyze the reduction of substrates such as acetylene and hydrazine, which the nitrogenases are also capable of reducing, the latter being one of the key intermediates in the reduction of atmospheric dinitrogen to ammonia.^25−27^ If FeMoco’s central structure is best described as two cubane groups bridged by sulfur atoms, then the [MoFe_3_S_4_]^3+^ cluster represents half the cofactor’s structure (see Figure 1), incorporating the Mo-containing cubane and with the interstitial carbide modeled by an additional sulfur atom. The [MoFe_3_S_4_]^3+^ cluster has been crystallized, and its structure matches the features of FeMoco well. Moreover, its electronic structure was calculated and found to be described by mixed-valent iron centers and a non-Hund rule Mo electronic configuration with a ground spin state of 1.5, which are electronic features it shares with FeMoco.^28−34^ Furthermore, a range of different variations of these complexes have been synthesized, which, alongside a more recently reported synthetic technique for precise control of core ligands in a given cluster, potentially allows for the tailoring of these complexes to fit reactions of interest.^35,36^

Experimental studies were performed to compare the relative hydrazine reduction activities of these molybdenum and vanadium cubanes and detected lower activity for the vanadium counterparts,^37,38^ as observed with the nitrogenases.^2^ However, unlike FeMoco, in FeVco the ground spin state and oxidation states of the metal ions are under discussion. The general consensus on the nitrogenase is that FeVco shares the same electronic structure and ground state as FeMoco (*S* = 3/2) with valencies [V^3+^, 3Fe^3+^, 4Fe^2+^].^6^ However, it should be noted that a recently published paper has proposed an alternative configuration, with an integer ground spin state and valencies on the transition metal elements [V^3+^, 4Fe^3+^, 3Fe^2+^] based on new EPR evidence.^39^ In its resting state, the structure of our vanadium complex in this study would be equivalent to the latter, as the [VFe_3_S_4_]^3+^ complex naturally has one fewer electron than its molybdenum counterpart.

From our own previous work, as well as prior literature, we confirmed that antiferromagnetic coupling within these complexes serves as an important factor in determining their geometries and electronic structures, and in turn, their reactivities.^40−42^ In that study,^40^ we determined that the lowest energy electronic states (with the broken symmetry density functional theory (DFT) solutions to describe antiferromagnetic coupling) of the relevant complexes were Ms = 1.5 for the molybdenum, and Ms = 1 for the vanadium. For all of the structures involved in the reaction pathways investigated, the spin and charge population analyses were closely monitored as part of verifying convergence to the correct electronic structure and to complement the data subsequently obtained from intrinsic bonding orbitals (IBOs). On the whole, the data supported the observations from the IBOs and further illustrated both the relative degree and importance of electron delocalization across the metal centers in each complex. The most notable example of electron delocalization in the system is between the Mo and Fe centers. Similarly to active species of the heme enzyme cytochrome P450 Compound I,^43−47^ the [MoFe_3_S_4_]^3+^ cluster adopts a non-Hund electron configuration with a doublet spin state with three unpaired electrons; in this case, with one α and two β electrons, the antiferromagnetic coupling interaction is optimized toward the two α iron centers and the one “flipped” β iron center. The strength of this interaction effectively pulls the iron atoms closer to the molybdenum, causing the distorted shape of the cubane, which is not observed if one were to geometrically optimize a high-spin configuration complex. This effect and interaction was observed in the vanadium complexes as well, albeit not quite to the same extent. The two iron atoms with unpaired α-spin electrons are mixed valence and equally share one additional β electron between them.

While molybdenum nitrogenase is better at reducing atmospheric nitrogen, which was part of the inspiration for our previous study,^40^ it is also experimentally known that vanadium nitrogenase is a lot more effective at binding and reducing carbon monoxide.^48,49^ Both Mo and V nitrogenases are capable of producing hydrocarbons from carbon monoxide, but the latter has been reported to be better at it by a factor of ≈800. The products of CO reduction by Mo-nitrogenase include C_2_H_4_, C_2_H_6_, C_3_H_6_, and C_3_H_8_, while vanadium also includes CH_4_ and some slightly longer-chain hydrocarbons. C_2_H_4_ is overwhelmingly the major product of these reactions, however, accounting for 94% of the hydrocarbon yield in the case of the vanadium nitrogenase.^48,50^

The currently industrially employed technique for the conversion of carbon monoxide to hydrocarbons is known as the Fischer–Tropsch process, which utilizes metal catalysts such as iron and cobalt under conditions of elevated temperature and pressure.^51,52^ Although the study of the mechanism of CO reduction by the nitrogenases has so far been limited largely to computation,^53,54^ studies into the binding of CO to the vanadium cofactor have concluded that binding can occur in both terminal and bridging configurations, with up to two CO molecules binding (one bridging, one terminal) as would be expected for formation of C2-products and beyond, with the bridging CO likely being the target of initial mechanistic steps due to its greater degree of activation.^50,55−59^ While such binding seems to occur in place of the bridging sulfur of the cofactors, which cannot be replicated by single cubanes, capability of CO reduction catalysis has been experimentally demonstrated on Fe_4_S_4_ single cubanes as well as synthetic derivatives of the cofactors (like MoFe_5_S_9_ and Fe_6_S_9_), indicating that the belt sulfur site, external protein environment, and interstitial carbide are not prerequisites for successful CO reduction in biomimetic catalysts.^60−63^ And while to date there has been no experimental data confirming the catalysis of CO reduction by the specific single cubanes chosen as the subjects for this study, by considering how a complex such as this might bind and activate CO in detail, we may gain insight into the structural features of potential catalysts for the production of hydrocarbons. Furthermore, certain features of the mechanism, such as the Mo/V–Fe interactions with the substrate and each other, could in turn provide part of the potential justification for the observed discrepancy between the Mo/V nitrogenase CO reduction efficacy.

## 2 Methods

The computational software package ORCA^64^ version 5.0.3 was used for all calculations. DFT calculations were done using the BP86^65^ functional for geometry optimizations followed by TPSSh^66^ single point calculations. All calculations included the D3BJ^67,68^ dispersion correction. The Ahlrichs def2-TZVP^69^ basis set was used on all atoms.

Broken symmetry solutions were found by first converging to a high spin solution of the cluster (e.g., for starting [MFe_3_S_4_]^3+^ complexes, Ms = 17/2 for Mo, 16/2 for V), then selectively flipping the spin on an iron atom with ORCA’s flipspin function (for a final Ms of 1.5 and 1.0, respectively) and optimizing on the broken symmetry potential energy surface.

The Conductor-like Polarizable Continuum Model (CPCM) with the SMD model^70^ was used to implicitly describe solvation in acetonitrile. Vibrational frequency analysis was performed on all structures in order to confirm the ground and transition states as well as to calculate Gibbs’ free energies (*T* = 298 K). The reduction and protonation free energy differences were reported relative to the calculated redox energy of cobaltocene and the deprotonation energy of lutidinium acid, respectively, to be used as a frame of reference.

Relaxed surface scans were used extensively to probe the feasibility of dissociation of certain species and transitioning between intermediates. In the relaxed surface scans reported in this study, the complex is geometrically optimized for each given length of a bond/interatomic distance of interest, always in increments of 0.1 Å. Where applicable and possible (e.g., for the dissociation of water), the highest energy point in a scan is then followed up by a transition state optimization.

IBOView^71^ software was used to localize the molecular orbitals produced by ORCA with the BP86 functional and view the resulting intrinsic bond orbitals (IBOs), which are generated with the software’s default settings. IBOs were plotted to gain insight into the electron flow and configuration changes during the overall reaction mechanism. These IBOs give quantitative insight into molecular orbital interactions and the degree of push–pull effects between partners, although the degree of interaction may be dependent on the computational method and basis set.^72,73^

## 3 Results and Discussion

### 3.1 Substrate Binding

#### 3.1.1 Initial Binding of a Single CO Molecule to (Mo/V)Fe_3_S_4_ Clusters

The capability to bind and activate the substrate of interest is, of course, the first major point of importance when these complexes. To that end, we first tested the binding energies of carbon monoxide to the potential metal binding sites within the complex. While several orientations of binding were tested, the only form that is stable after geometry optimization was the terminal M–C–O form. When we attempted other configurations such as side-on binding or binding in a bridging manner between two different Fe centers, it led to an optimized geometry with the terminal configuration. The studies of the binding of CO to the nitrogenase cofactors have proposed the presence of a bridging μ-CO to be the primary site of CO binding to the cofactors; however, this would occur in the space between two different cubanes rather than on one of the cubanes’ faces,^50,74^ which is not a binding mode that can be replicated by the complex studied here. The side-on binding mode has been proposed specifically for the studies of the Fischer–Tropsch mechanism where it would facilitate dissociation of CO to M–C and M–O species and to facilitate C–C bond formation.^51^ Furthermore, the geometric orientation matches previous studies on CO-bound iron complexes well.^53,75,76^

The binding energies of the CO substrate on the metal centers in the end-on configuration are given in Table 1. Binding of CO to either Mo or V leads to replacement of an acetonitrile ligand and gives the iron atom a 5-coordinate orientation with trigonal bipyramidal configuration that has the CO in the axial ligand position. The binding is favorable to Mo/V centers on all complexes, with binding free energies starting at just under neutral for the isocharged vanadium complex to just under −10 kcal mol^–1^ for the molybdenum complex. When it comes to the Fe binding sites, however, the CO is only able to bind favorably to the Fe centers in the vanadium complexes, and more favorably than on the vanadium binding site at that. The unfavorable binding to Fe compared to Mo on the molybdenum clusters agrees with experimental evidence for similar cubane clusters.^77,78^ We note that the unfavorable binding energy for Fe3 is due to that site being the chosen “flipped” spin site for our broken symmetry solution, and binding of the CO on that particular site serves to disrupt the (anti)ferromagnetic coupling interactions.

**Table 1:** Δ*G* Binding Energy of Carbon Monoxide to the Studied Complexes at the Given Centers

Δ*G*/kcal mol^–1^				
complex	Mo/V	Fe1	Fe2	Fe3
[MoFe_3_S_4_]^3+^	–9.4	11.2	11.2	11.0
[VFe_3_S_4_]^2+^	–5.5	–6.5	–9.8	6.8
[VFe_3_S_4_]^3+^	–0.3	–2.0	–7.2	1.2

Activation of CO requires an extent of backbonding to the ligand in order to introduce electron density to the π* orbital, weakening the C–O bond. The simplest way of probing the extent of CO activation is by looking at the CO bond lengths in the optimized structures, as well as their associated IR stretch vibrations, which are presented in Table 2. The data in Table 2 shows an inverse relationship between the C–O bond length and the C–O stretch vibration, where a short C–O distance, e.g., in [MoFe_3_S_4_]^3+^ (row 1) of 1.145 Å corresponds to the largest vibrational frequency of the series. As such, the C–O vibrations correlate well with Badger’s law of the inverse link between bond length and bond vibration.^79^ By considering the resting carbon monoxide bond length of around 1.13 Å and typical C=O double-bond lengths of 1.16–1.21 Å,^80^ the molybdenum complex with its Mo binding site is able to only slightly activate the CO, while the vanadium complexes on their iron binding sites can achieve a bond length within the range of a typical C=O double bond. The ν_CO_ value of the latter is consistent with those typically associated with the terminal CO ligands in experiments on the V-nitrogenase’s VFe protein, but unsurprisingly falls short of the μ-CO value of ≈1720 cm^–1^.^81^ Other synthesized iron–sulfur complexes with enhanced CO activation have been shown to have C–O stretch vibrations in the range of 1851–1832 cm^–1^ (and as low as 1782 cm^–1^ when considering reduced form with countercation).^61,82^

**Table 2:** Optimized Carbon–Oxygen Bond Lengths When Bound to the Different Complexes as well as the Associated IR Stretch Frequency

complex	site	bound CO bond length/Å	ν_CO_/cm^–1^
[MoFe_3_S_4_]^3+^	Mo	1.145	2023
[VFe_3_S_4_]^2+^	V	1.152	1968
[VFe_3_S_4_]^3+^	V	1.148	2007
[MoFe_3_S_4_]^3+^	Fe	1.158	1937
[VFe_3_S_4_]^2+^	Fe	1.172	1884
[VFe_3_S_4_]^3+^	Fe	1.165	1931

**Figure 2:**
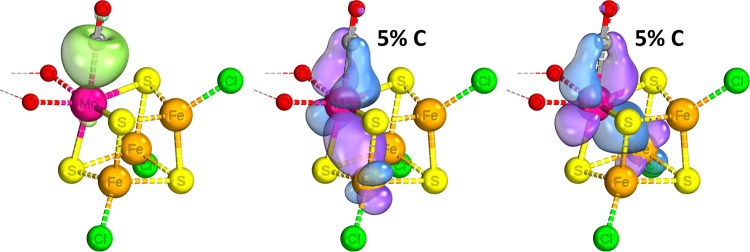
IBOs involved in Mo–CO binding for the [MoFe_3_S_4_]^3+^ complex, with the percentage indicating the degree of localization of the orbital shown on the carbon center. The iso-surface threshold was taken as 80%.

**Figure 3:**
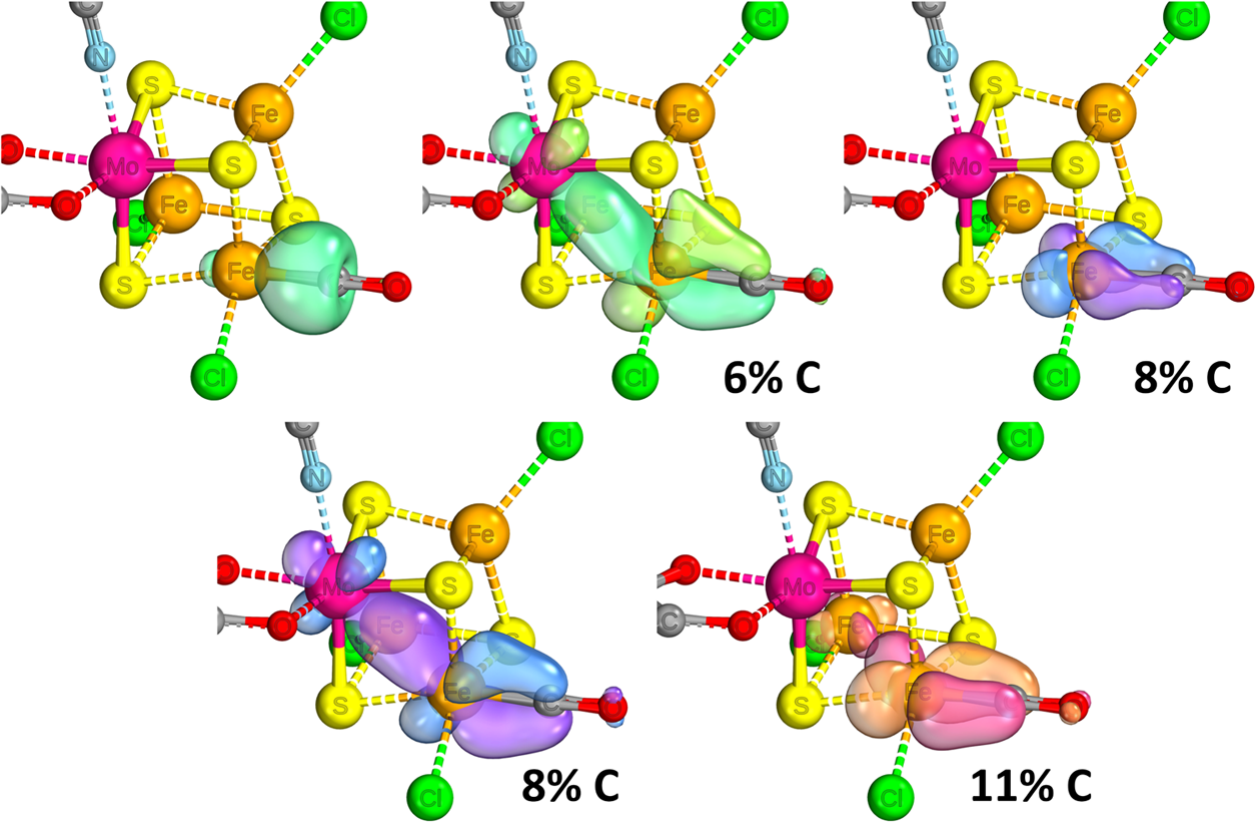
IBOs involved in Fe–CO binding for the [MoFe_3_S_4_]^3+^ complex, with the percentage indicating the degree of localization of the orbital shown on the carbon center. The iso-surface threshold was taken as 80%.

**Figure 4:**
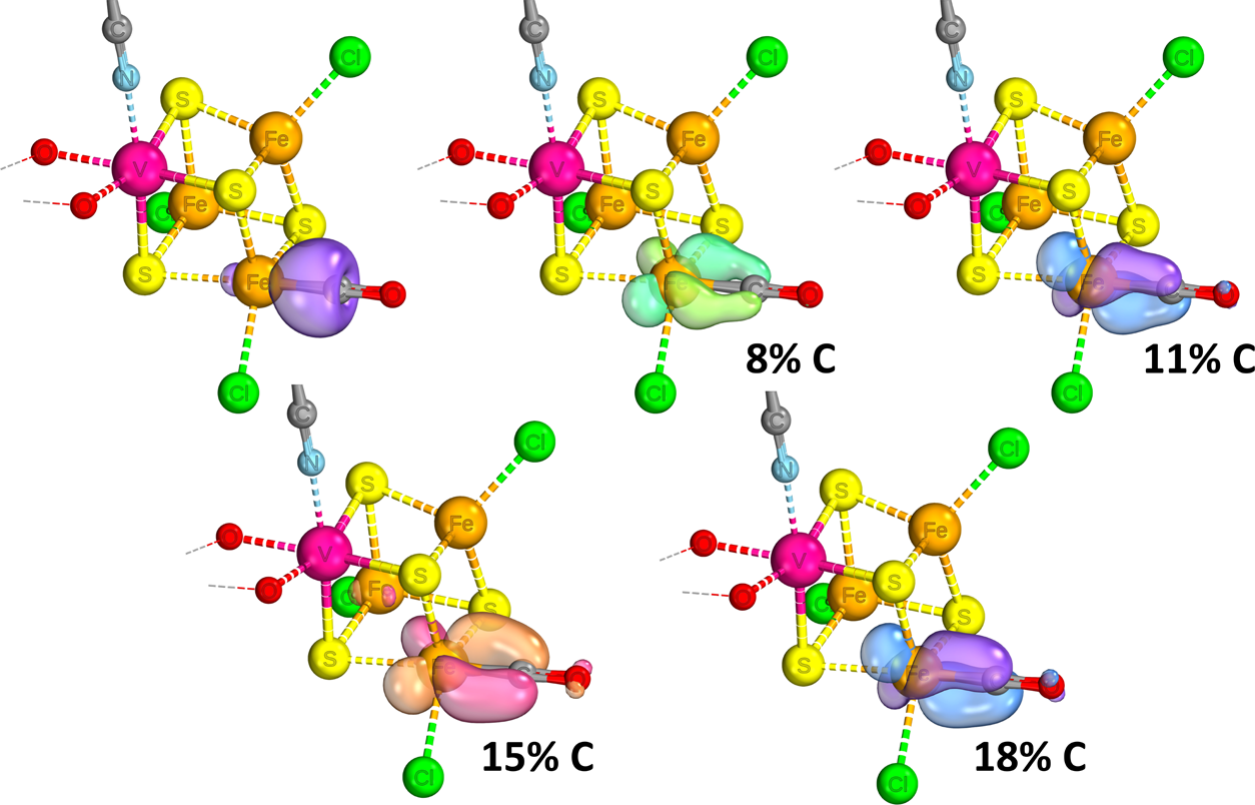
IBOs involved in Fe–CO binding for the [VFe_3_S_4_]^2+^ complex, with the percentage indicating the degree of localization of the orbital shown on the carbon center. The iso-surface threshold was taken as 80%.

To investigate the binding and backbonding in more detail, we looked at localized intrinsic bond orbitals (IBOs) of the optimized structures. The IBOs for M–C bonding interactions are shown in Figures 2–4. The backbonding from the Mo/V binding sites involves only 2 electrons, which explains the lesser degree of CO elongation for the Mo/V binding sites compared to the iron. As can be seen in Figure 2, the two electrons continue to be significantly involved in the Mo–Fe coupling interactions, providing only 5% of each orbital’s electron density back to the ligand. Binding on the Fe site in the Mo complex is able to involve a larger number of electrons in the backbonding and, furthermore, with each electron being more strongly localized to the carbon center than when bound to Mo. Comparing the cases of Fe binding to isoelectronic Mo and V complexes (Figures 3 and 4, respectively), the extent of backbonding in the latter is notably higher. The best explanation we can infer from the IBOs is that while the stronger Mo–Fe interactions are maintained despite binding of CO, the vanadium complex is able to forego the relatively weaker V–Fe interactions in favor of more strongly binding the CO. Essentially, in the molybdenum complex, the antiferromagnetic coupling interactions compete more strongly with the π-backbonding to the CO, which explains its relative lesser extent of CO activation. Molybdenum’s stronger interaction with neighboring iron centers is something that has also been reported in computational studies on models of the full molybdenum and vanadium cofactors,^41^ so this effect may play a part in lowering the FeMoco’s CO reduction efficacy.

Looking at the electronic state of the isoelectronic (i.e., resting state Ms = 1.5) Mo/V complexes as a whole, upon binding of the CO, the Fe center in question adopts a formal oxidation state of +2 (as opposed to mixed valence +2.5 in its resting state), with 4 α and 2 β electrons rather than 5 α and 0.5 β electrons. This change in electronic state occurs in order to enable π-backbonding to the CO with two pairs of electrons, as shown on the IBOs previously. As a result, the lowest energy electron configuration of the complex becomes Ms = 0.5 with the Mo/V centers maintaining their configurations with ββα and ββ, respectively. An alternative electronic configuration calculated to retain the original Ms = 1.5 spin state was found to only be 1.2 kcal mol^–1^ above the ground state for the vanadium complex; however, this still maintains the same +2 configuration on the iron but with an αβ electron configuration on the vanadium instead. This change in Fe electronic configuration and hence overall ground spin state upon binding of CO is consistent with experimental data regarding CO binding to Fe centers on other systems,^83,84^ as well as specifically the reported change in EPR signal from *S* = 1.5 to 0.5 under CO turnover conditions in FeMoco.^74,85^

#### 3.1.2 Binding of Second CO

As the reduction of CO by nitrogenases largely results in products with 2 or more carbon atoms, all of which have been previously shown to originate from the inbound CO gas under turnover conditions through ^13^C-labeling,^50^ the simultaneous binding of multiple substrates would be required. We have found that the binding of a second CO could happen at many points along the studied mechanism with the preferred binding site being the same Fe center as the initial one. This seems to be largely due to the binding of another ligand enabling the formation of a pseudo-octahedral geometry around that Fe and assumption of a low spin d^6^ electronic state. Furthermore, binding of CO on alternative Fe centers would require the assumption of multiple lower spin d^6^ iron centers, which is either implausible with certain electron configurations (such as resting state [VFe_3_S_4_]^3+^) and/or would necessitate weakening the antiferromagnetic couplings within the complex to a greater extent than maintaining the configuration on one center only. In the case of the 3+ vanadium complex, the lowest energy electronic state with CO bound ends up being Ms = 0 with V(αβ) Fe1(5α) Fe2(3α3β) Fe3 (5β), while in the case of the 2+, Ms = 3.5 with V(2β) Fe1(5α)-β-Fe3(5α) Fe2(3α3β). The Ms = 0.5 configuration for the latter with V(2α) Fe1(5αβ) Fe2(5β) Fe3(3α3β) was calculated to be only 1.4 kcal mol^–1^ higher in energy. On the whole, a d^6^ Fe center seems best suited to bind a CO substrate, and the binding of a second CO to the same Fe center merely requires it to switch from a pseudo-high-spin to low-spin d^6^ configuration; binding of a CO to an alternate Fe center, on the other hand, would not be able to be stabilized by a d^6^ Fe center, as the electron configuration of the complex could not reasonably permit the formation of two d^6^ Fe centers. While the formation of two d^6^ Fe centers is plausible in the singly reduced molybdenum and its isoelectronic vanadium counterpart, this is still unfavorable, most likely due to the greater disruption of the (anti)ferromagnetic coupling of the complex, as opposed to binding on the same Fe center. This observed evolution of the oxidation and ground spin state for the coordinatively unsaturated Fe center upon binding of consecutive CO ligands follows precedent in the literature; for example, one study reported a shift in the spin state of a 4-coordinate Fe(II) complex from *S* = 2 to *S* = 1 upon binding of CO,^86^ while another reported a change in both the oxidation and spin state of a 4-coordinate Fe(III) complex to Fe(II) with spin state shifting from *S* = 5/2 to *S* = 1.^87^ Furthermore, there are several examples of four- and five-coordinate Fe complexes that adopt a singlet configuration upon binding of CO ligand(s).^83,88,89^

#### 3.1.3 Binding of CO_2_

**Figure 5:**
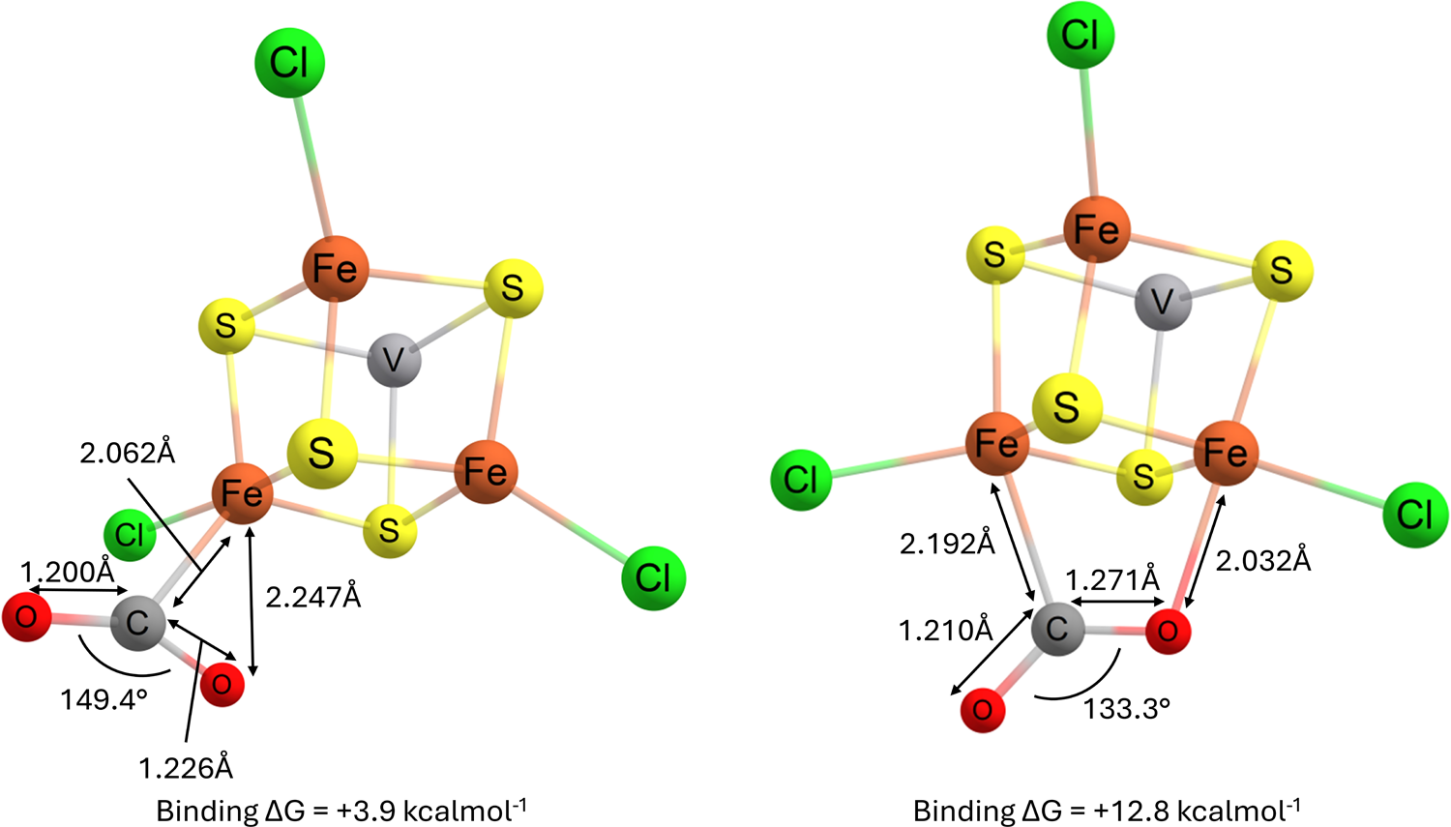
Geometry-optimized local minima for the binding of CO_2_ to the [VFe_3_S_4_]^3+^ complex alongside the calculated free energy change upon binding.

Carbon dioxide is another known substrate of the nitrogenase enzymes, and shown to produce products on wild-type nitrogenases including formate, methane, and CO.^90^ As the CO_2_ substrate could potentially serve as a precursor to the rest of the mechanism, we considered the binding of CO_2_ to the complexes. While we were able to find and optimize geometries where CO_2_ would stay bound, which are shown in Figure 5, the calculated free energy difference upon binding indicates that CO_2_ would not bind to this complex under the modeled conditions.

Nonetheless, the most stable binding mode of CO_2_ to this complex is through a bent conformation via the carbon, which agrees with previous computational studies on iron systems,^75,76,91^ as well as proposals by structural and computational studies on the nitrogenase itself. As those studies conclude that the active site for the binding and reduction of CO_2_ in the nitrogenases does not take place in the Mo/V-containing cofactors, but rather in a different [Fe_4_S_4_] cluster elsewhere in the enzyme, it is not surprising to see the binding free energy Δ*G* calculated as unfavorable. In addition, an external feature that can stabilize the bent conformation of the substrate, such as through H-binding to an -NH_2_ group of Arginine, may be required in order to make such binding favorable.^90,92,93^

### 3.2 Reaction Mechanism

#### 3.2.1 First Protonation and Reduction—Fe Binding

An isolated CO molecule has a dipole moment that gives a partial positive charge on the oxygen atom, which will prevent it from protonation easily. Moreover, the carbon atom of CO lacks lone pairs when it is bound to a metal center. Therefore, in order to overcome the first major hurdle in the mechanism and proceed with the first protonation, the metal-CO group must be provided 2 electrons in order to allow it to form a metal–carbonyl, feasibly enabling the protonation of either the carbon or oxygen centers.

**Figure 6:**
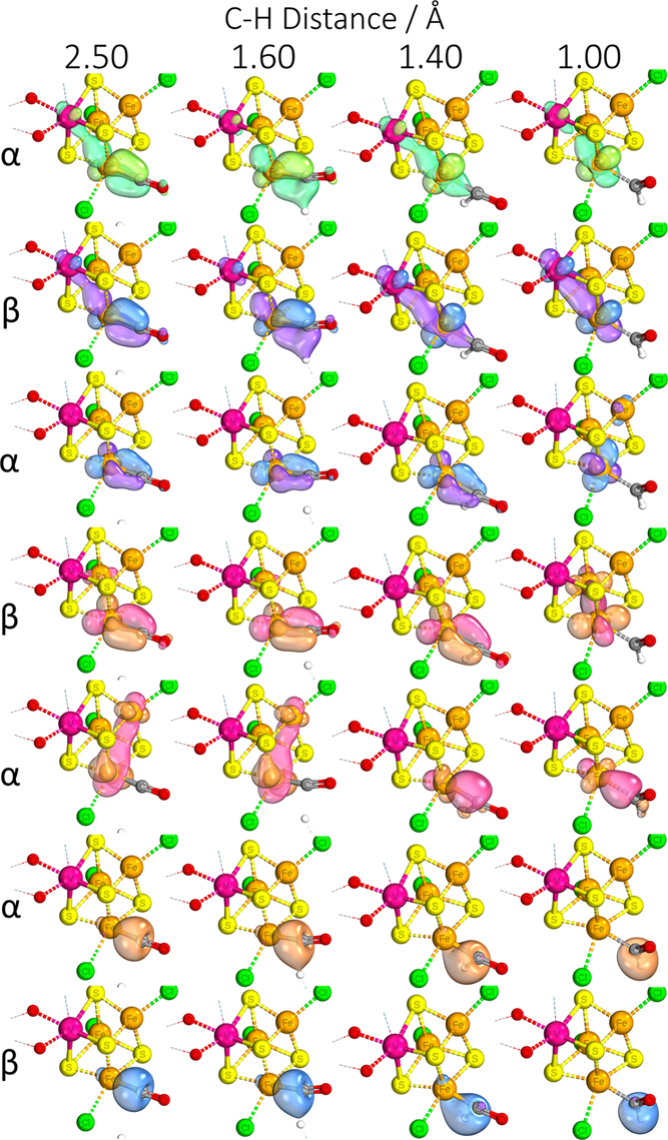
IBOs that display significant change over the course of a C–H relaxed surface scan, showing electron movement during the protonation of the CO substrate’s carbon center when bound to the [VFe_3_S_4_]^2+^ complex. The iso-surface threshold was taken as 80%.

**Figure 7:**
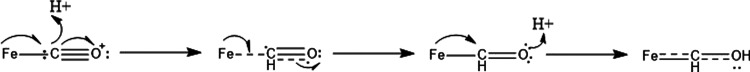
Mechanism of CO protonation/reduction showing the first two protonation steps after binding on the Fe center of the [VFe_3_S_4_]^2+^ complex.

As indicated in Figures 3 and 4, binding of the ligand to the Fe center is accompanied by a four-electron backbonding interaction. Figures 6 and 7 illustrate the movement of electrons during protonation; these backbonding interactions largely break down (rows 1–4), with one of the electrons from the C–Fe dative covalent bond/lone pair and one of the iron’s backbonding electrons moving to capture the proton (rows 1/2 and 6/7). The substrate remains bound to the iron center with 1 electron as a radical, and the CO bond transitions to a double bond with a slight triple bond character. At the same time, and for any other mechanistic steps involving the transfer of an electron to the substrate, a valence electron from the Fe–S network moves to “replenish” the Fe center in question (row 5). As can be seen from the IBOs, this initial protonation is further driven by the restoration of the V–Fe interaction which was suspended upon initial substrate binding in order to accommodate backbonding.

**Figure 8:**
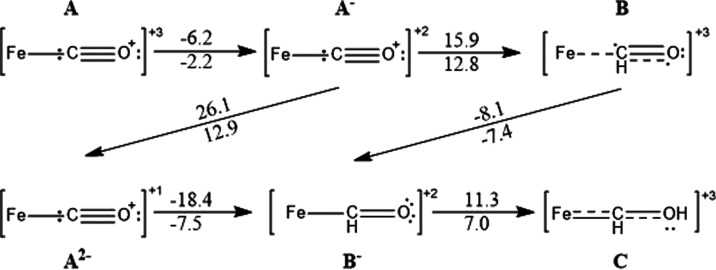
Thermodynamics of sequential and staggered electron and proton transfer to [VFe_3_S_4_]^3+^ for systems with (top) and without (bottom) acetonitrile bound. Free energies are given in kcal mol^–1^ and include zero-point, thermal, and entropic corrections at 298 K.

Starting from the isocharged complexes, the first protonation event could feasibly occur after one or two instances of reduction of the complex. Protonation only becomes exergonic (relative to reference) after the addition of 2 more electrons to the complex. This is easier to accomplish for the molybdenum complex than the vanadium; as discussed in the previous study,^40^ vanadium has a notably lower tolerance for excess reducing equivalents/electron density being stored in the M–S network. That said, in the particular circumstance of a substrate binding on an iron center, the acetonitrile ligand on the Mo/V center is labile and capable of associating and dissociating with only a small barrier (can be as low as <1 kcal mol^–1^, depends on specific structure). Depending on the identity of the intermediate structure, it can be stabilized or destabilized by the presence/absence of the acetonitrile ligand, by up to ±10 kcal mol^–1^. This is potentially an exceptionally useful mechanistic feature; in the hypothetical experimental setting, the reaction being conducted in acetonitrile as a solvent would allow this exchange to occur very rapidly as required to stabilize a given intermediate, lowering reaction barriers. Meanwhile, in the case of the nitrogenase cofactors, in place of the acetonitrile is a Histidine residue (H442, resting state FeMoco PDB: 3U7Q)^94^ which is positioned to likewise potentially be able to bind/unbind from the Mo/V center as required for greater stability. The sequence of protonation and reduction events leading to the first protonated structures alongside the relevant calculated energies is depicted in Figure 8, also highlighting the difference between steps with acetonitrile present or absent. While a large number of potential intermediates were considered, many were omitted due to being vastly less favorable than other possibilities, such as in this particular case the protonation of the O center first, which is less favorable than that of carbon by ≈20 kcal mol^–1^.

#### 3.2.2 Single Substrate Reduction

**Figure 9:**
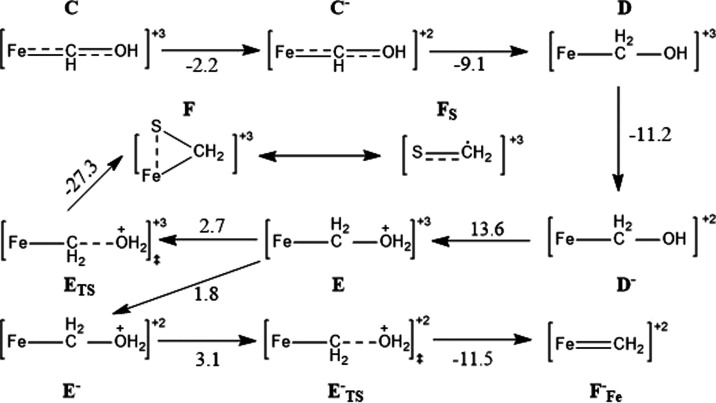
Further CO protonation/reduction steps from Fe–CHOH. The relevant free energies given are for structures with acetonitrile dissociated, and all energies are given in kcal mol^–1^.

**Figure 10:**
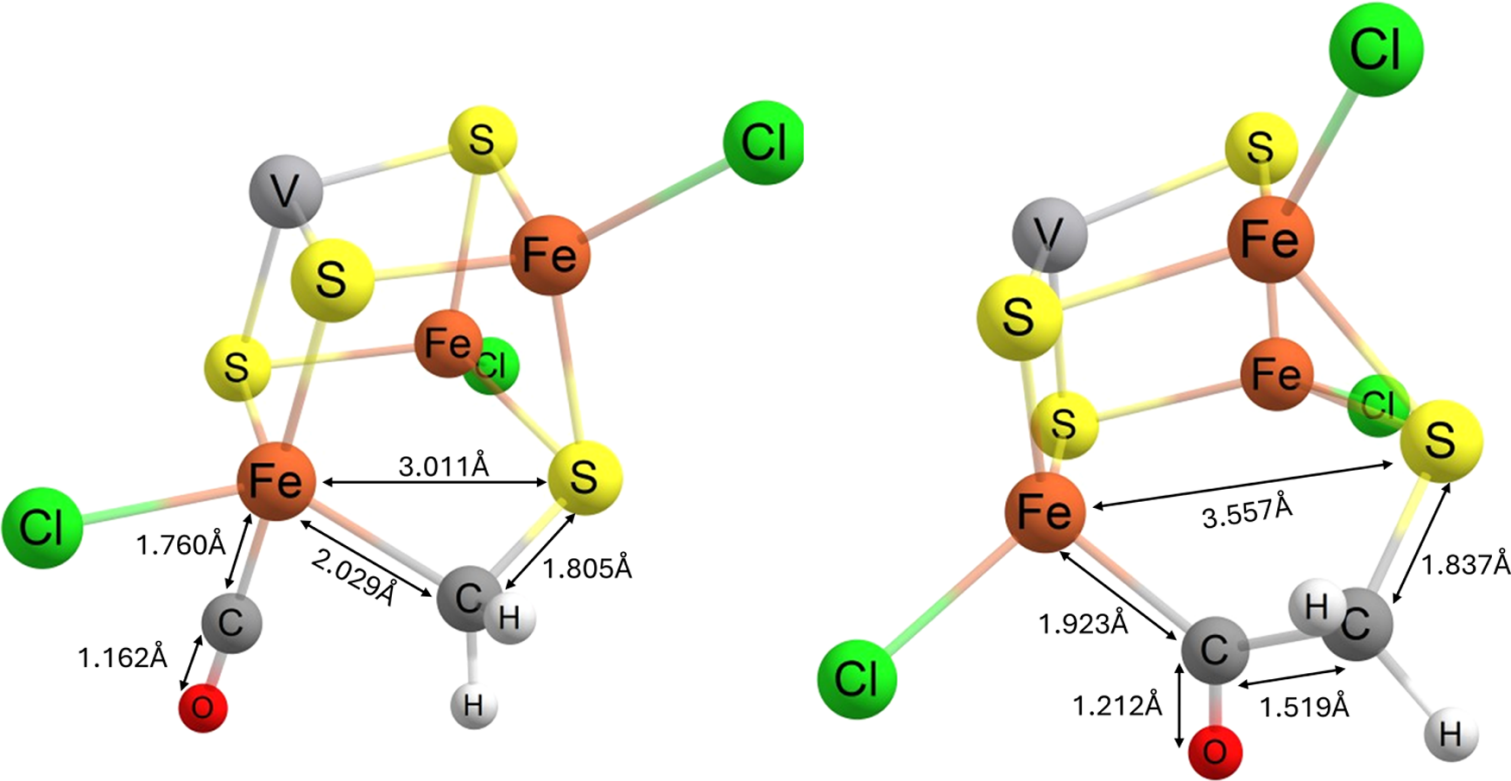
Structures of key intermediates before and after C–C bond formation via a “sandwiched” intermediate (**F**_CO_ → **G** on Figure 13). Parts have been omitted for clarity.

From the M–CHOH (**C**) complex, excluding the binding of a second CO, the subsequent mechanistic steps are summarized in Figure 9. The reaction most favorably proceeds via another reduction (exergonic by 2.2 kcal mol^–1^) followed by another protonation of the carbon center (exergonic by 9.1 kcal mol^–1^). A second protonation of the O center at this stage requires another electron reduction (exergonic by 11.2 kcal mol^–1^) followed by a barrier of 13.6 kcal mol^–1^ for protonation itself. The water can readily dissociate from this M–CH_2_OH_2_ (**E**) intermediate with a small transition state free energy barrier of 2.7 kcal mol^–1^. From here, the geometry optimization forces the rearrangement of the cluster to allow for the formation of an S–C covalent bond and the overall Fe–CH_2_–S intermediate (**F**) (see Figure 10). If the dissociation of water were to occur after another reduction (endergonic by 1.8 kcal mol^–1^ with transition state barrier of 3.1 kcal mol^–1^), a geometry whereby the substrate remains bound solely on the Fe can be achieved by optimizing on an Ms = 0.5 broken symmetry PES rather than Ms = 1.5, by switching to the previously discussed electronic configuration with the Fe center d^6^ for better stabilization of π-type interactions. While both of these intermediates could be reached without imposing special constraints or changing the starting geometry relative to the previous structure (other than removal of the water), the sandwiched intermediate [Fe–CH_2_–S]^2+^ (**F**^–^) has a calculated free energy lower than that of the [Fe = CH_2_]^2+^ (**F**_Fe_^–^) intermediate by 27.4 kcal mol^–1^. That said, as we cannot conclusively exclude either of the intermediates, both scenarios have been calculated and followed through for completeness.

Considering the sandwiched intermediate (**F**), to probe further reactivity, we have conducted relaxed surface scans of the Fe–C and S–C distances. Stretching of the Fe–C distance by 0.5–0.6 Å to a point where further protonation could be expected to occur requires overcoming a barrier Δ*E* = 10.7–13.5 kcal mol^–1^, while the equivalent for the S–C distance is 19.9–21.3 kcal mol^–1^. Scanning all the way back to the terminal configurations Fe–CH_2_ (**F**_Fe_) and S–CH_2_ (**F**_S_) requires >22 kcal mol^–1^ in both cases. If bonded to sulfur exclusively/terminally, the CH_2_ species is a radical, with the sulfur–carbon bond adopting a partial (3 electron) double bond. Were these barriers to be overcome, an S/Fe–CH_3_ (**G**_sat_) species could be formed with the addition of another electron (−9.5 and −12.3 kcal mol^–1^, respectively) and proton (−25.5 and −24.6 kcal mol^–1^, respectively). Considering for the moment only the formation of CH_4_ as a product, further protonation of the substrate would require its full or (more likely) stretch/partial dissociation from the complex, which is more favorable following one last reduction, exergonic by 3.2 kcal mol^–1^ from the S variant and 2.0 kcal mol^–1^ from the Fe variant. While calculating the barrier for the full dissociation of the CH_3_ radical is difficult due to the radical, lengthening of the S–C bond by 0.4, 0.5, 0.6, and 0.7 Å has the CH_3_ adopt an increasingly planar-like geometry with barriers of 10.8, 13.3, 15.2, and 16.5 kcal mol^–1^, respectively (from a 2+ intermediate). The equivalent lengthening of the Fe–C distance costs 8.2, 11.0, 13.7, and 16.0 kcal mol^–1^ (the relaxed surface scan data in more detail can be found in the Supporting Information). Upon the final protonation, methane can be released, regenerating the catalyst to its starting geometry (to be precise, more likely its singularly reduced state [VFe_3_S_4_]^2+^). The reformation of the cubane Fe–S bond as a result of substrate dissociation serves to reduce the barrier for this final protonation.

While the sequence of events discussed above is the most straightforward way of arriving at CH_4_ as a product, a second CO substrate could have favorably bound at some point prior to the Fe–CH_2_OH (**D**) intermediate. We then attempted to proceed the mechanism from the OC–Fe–CH_2_OH (**F**_FeCO_) intermediate; however, the subsequent protonation gives an unstable, high-energy intermediate, likely due to the inability of a 6-coordinate Fe center to form a partial double bond to the −CH_2_. As a result, the only way to obtain the desired intermediate with a reasonable geometry and energy is to optimize from a prebroken Fe–S bond while constraining the Cl on the Fe center. With that in mind, in order for the reaction to proceed, the second CO substrate would need to dissociate (barrier of 6.4 kcal mol^–1^ from the Fe–CH_2_OH (**D**) intermediate) or the protonation would need to happen simultaneously with either the formation of a C–C bond or a sandwiched OC–Fe–CH_2_–S (**F**_CO_) intermediate. These relevant energies for this pathway, including the constrained intermediates, are provided for completeness in the Supporting Information. Assuming that the reaction toward CH_4_ would proceed with the second CO still bound, the subsequent steps from the OC–Fe–CH_2_–S (**F**_CO_) → S–CH_2_ (**F**_SCO_) intermediates are exergonic with 10.7, 15.4, and 10.1 kcal mol^–1^ for the next reduction, protonation, and another reduction, respectively, while from the Fe–CH_2_ (**F**_FeCO_) intermediate exergonic by −39.0 and −9.5 kcal mol^–1^ for protonation and reduction, respectively. The S/Fe–CH_3_ scans do not appear to be affected significantly by the presence of the second CO ligand.

#### 3.2.3 Formation of the Carbon–Carbon Bond and Subsequent Products

**Figure 11:**
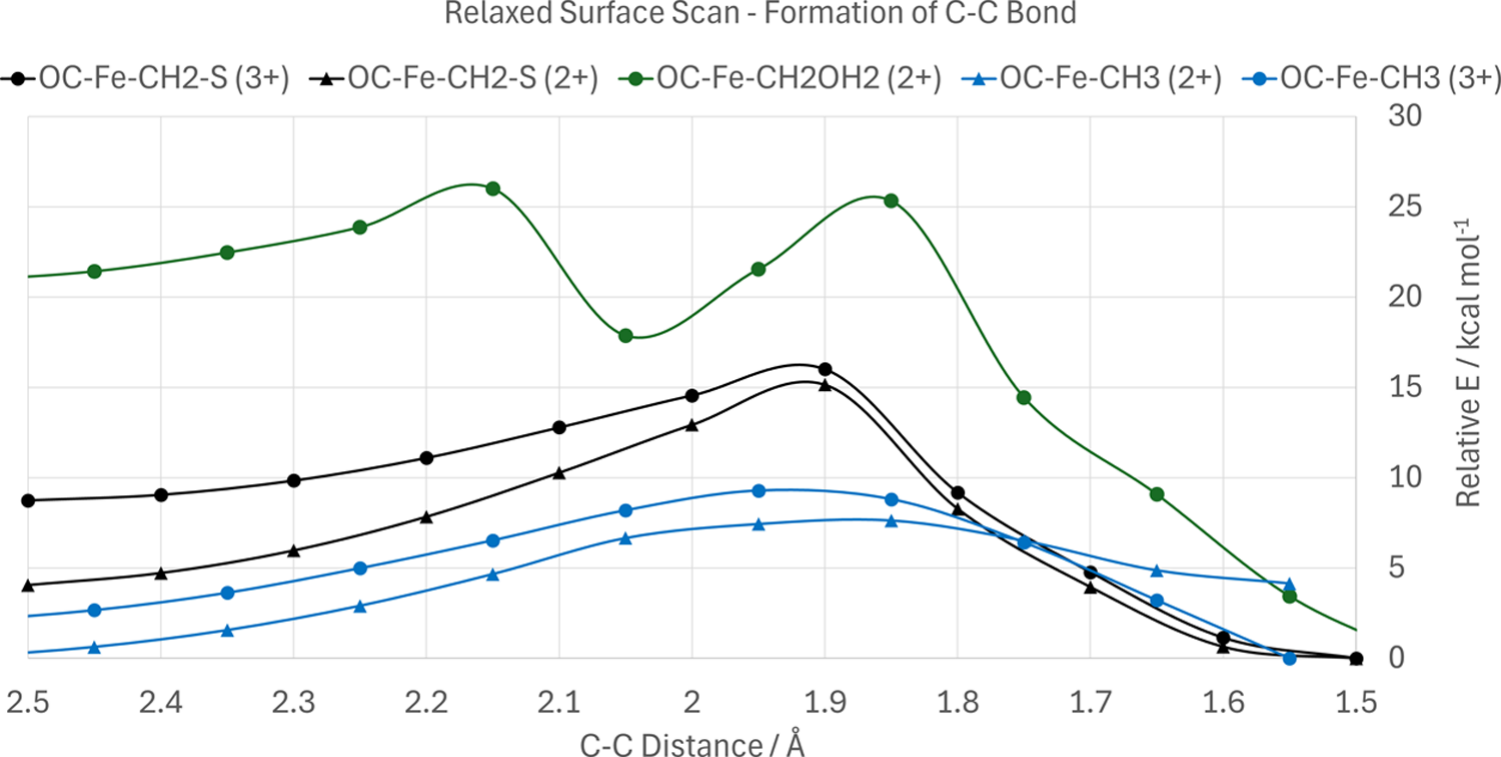
Relaxed surface scan graph, showing the relative energies of optimized structures for each fixed C–C distance. For the OC–Fe–CH_2_OH_2_ intermediate, the first saddle corresponds to the dissociation of water from the complex, while the second saddle corresponds to the C–C bond formation.

Subsequently, we investigated the formation of C2 hydrocarbons by forming a C–C bond between two C1 intermediates. We started with exploring the C–C bond formation between −CH_2_ and −CO. Even if the Fe–CH_2_–S (**F/F**_CO_) sandwich occurs, a prospective second substrate would remain in close proximity to the CH_2_, and a shortening of the OC–CH_2_ distance in such an intermediate results in an overall exergonic reaction after a barrier of 7–11 kcal mol^–1^ depending on the electron count, which is shown in Figure 11. If the secondary CO ligand is already bound on the iron center at the time of the dissociation of the water byproduct, the barriers to both the dissociation and formation of the CC bond are lessened, as the former experiences an additional driving force while the latter additionally starts at a closer distance. The relaxed surface scan barriers starting from the OC–Fe–CH_2_ (**F**_FeCO_) intermediate are only 5.1 and 1.7 kcal mol^–1^ for the C–C bond formation and H_2_O dissociation, respectively. If occurring from the “sandwich” (**F/F**_CO_) intermediate, such as may be in the case of the secondary CO ligand not being nearby at the time of H_2_O dissociation, the radical (or lone pair, depending on the electron count of the complex) of the CH_2_ can attack the carbon of the bound CO, with the Fe center donating another electron in the former case. The new C–C bond would therefore form via the removal of the Fe-CH_2_ bond while maintaining the Fe–CO bond throughout. This forms a Fe–COCH_2_–S (**G**) species if initiated from an intermediate with a S–C bond, or either a Fe–COCH_2_/Fe–COCH_2_–S (**G**_Fe_/**G**) species otherwise. The geometries before and after C–C bond formation via the “sandwiched” intermediate are depicted on Figure 10, with the corresponding electron structure changes across the transition visualized with IBOs onFigure 12.

**Figure 12:**
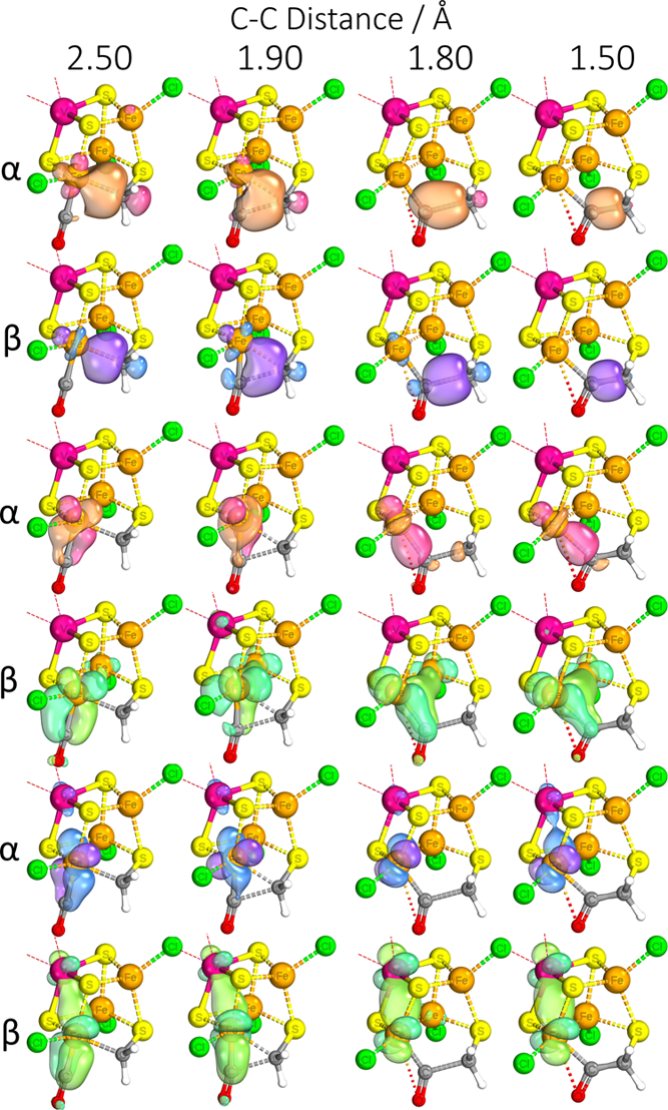
IBOs that display significant change over the course of a C–H relaxed surface scan, showing formation of a C–C bond between the −CO and −CH_2_– species when bound to the [VFe_3_S_4_]^3+^ complex. The iso-surface threshold was taken as 80%.

From the IBOs, it is easy to see that due to the two substrates’ relative positions as cis ligands, they are perfectly prepositioned to initiate the formation of the C–C bond (rows 1–2). The extent of the Fe–C backbonding weakens following the formation of the C–C bond, which once again would serve as a way to drive the reaction forward as the V–Fe and Fe–Fe interactions are re-established/strengthened as a benefit of C–C bond formation (rows 3–6). That said, some extent of Fe–C backbonding remains, which serves to weaken the C=O bond for the subsequent protonation step (row 4). Formation of a C–C bond from an Fe–CO/S–CH_3_ intermediate was not possible, with a barrier of ≈50 kcal mol^–1^, but was achievable from a OC–Fe–CH_3_ (**G**_COsat_) intermediate, with a barrier of 7.3–7.6 kcal mol^–1^ depending on electron count, resulting in a Fe–COCH_3_ (**H**_sat_) intermediate. Proton transfer resembling enol-keto tautomerism does not occur at this stage, with relaxed surface scans showing massive barriers of >60 kcal mol^–1^. The relaxed surface scans showing C–C bond formation are summarized in Figure 11.

**Figure 13:**
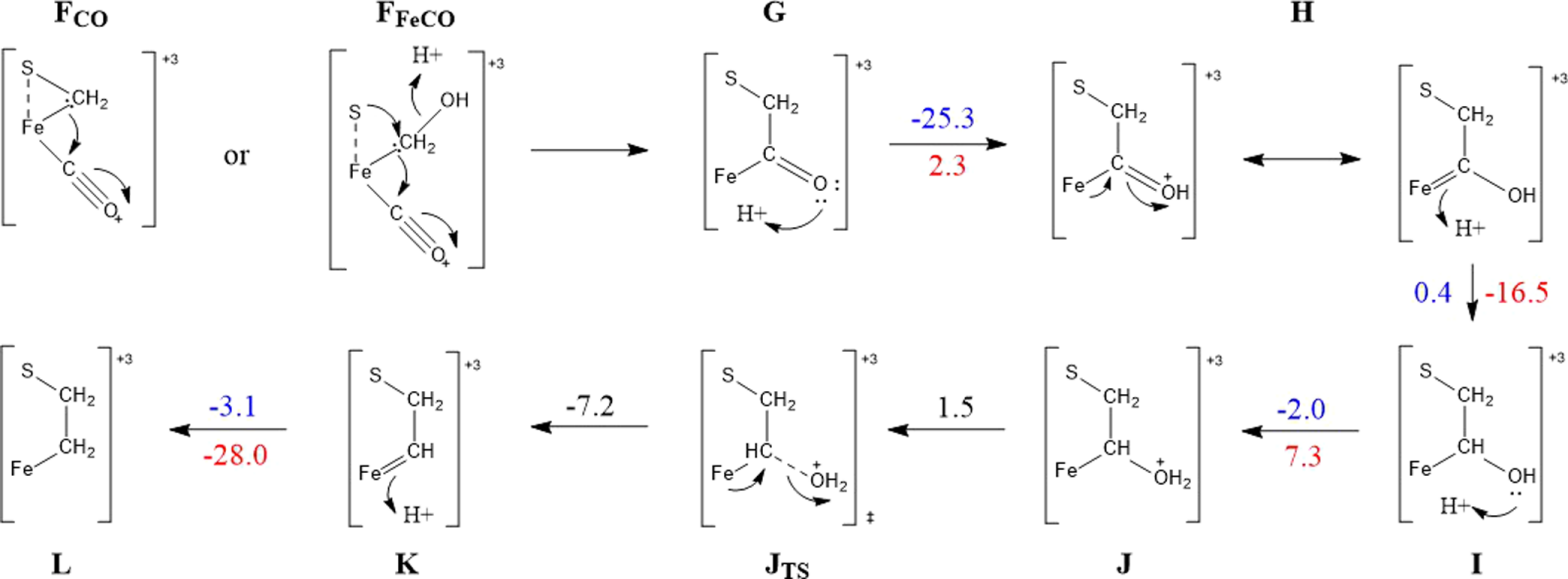
C–C bond formation and further protonation/reduction mechanistic steps from OC–M–CH_2_–S, also showing extensive charge delocalization through resonance structures. The relevant energies given are for structures with acetonitrile dissociated, and are given in kcal mol^–1^. The energies are provided in blue for reduction steps and in red for protonation steps, which occur in the order of reduction first.

**Figure 14:**
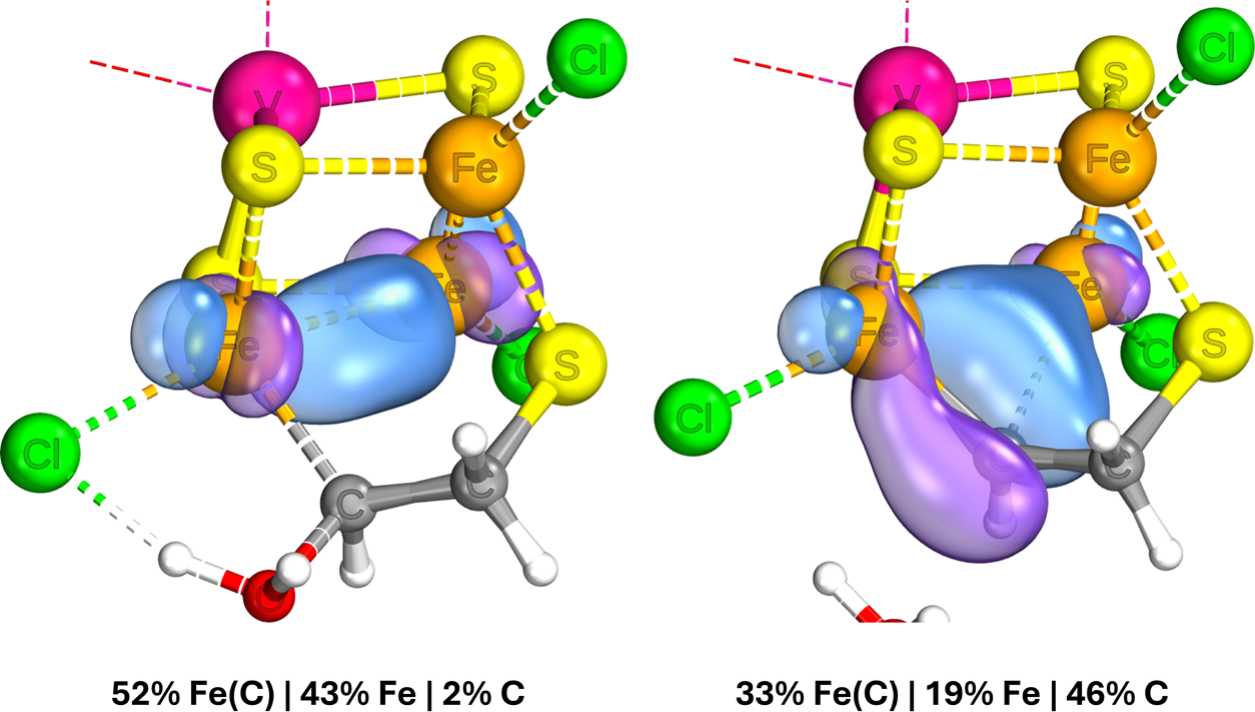
IBOs showing the mixed-valence Fe–Fe β electron before and after the dissociation of H_2_O from the Fe–CH(OH_2_)CH_2_–S intermediate. The iso-surface threshold was taken as 80%.

The steps of C–C bond formation and the most favorable sequence of following protonation/reduction steps are summarized in Figure 13. A route of resonance-based stabilization is shown, and IBOs indicate that the primary method of stabilization of the intermediate continues to be primarily the Fe–C interaction with very strong backbonding, to the extent where it is akin to a proper π-bond. It is also worth noting that the proton on the oxygen aligns itself as close as possible to the nearby chloride ligand for a further stabilizing electrostatic interaction, which in turn also allows the oxygen center to continue maintaining a significant double-bond character toward the carbon center. For the same reason, although both the carbon and oxygen centers could feasibly be further protonated at this stage, protonation of the carbon center (**H** → **I**) is more favorable by a very wide margin of 26.0 kcal mol^–1^. Once that occurs, the double-bond character between the carbon center and its neighbors ends, and the oxygen can be protonated next (**I** → **J**). This seems to be the most difficult/rate-limiting step of the reaction when considering the steps occurring after the formation of the C–C bond. The water molecule readily dissociates with a transition state free energy barrier of 1.5 kcal mol^–1^ (**J**_TS_), once again enabled by the iron center’s formation of a pseudo-double bond to the carbon. More specifically, a component of this interaction is the mixed-valence electron between the up spin iron centers also becomes strongly localized on the carbon center, creating in effect a partial bond between the carbon center and two iron centers. The IBOs from before and after the dissociation illustrating the delocalization in question can be seen in Figure 14. The subsequent protonation and reduction steps occur readily with free energies of −3.1 and −28.0 kcal mol^–1^ for reduction and protonation, respectively, ending up with a CH_2_–CH_2_ (**L**) species, which is a natural point of consideration for the release of the stable molecule CH_2_=CH_2_. Release of ethylene can occur through the near-concurrent formation of the C=C double bond and the reformation of the cubane/Fe–S bond, which is best observed by scanning the Fe–C distance; while calculation of the exact transition state geometry proved difficult, the relaxed surface scan showed a barrier Δ*E* = 12.9 kcal mol^–1^, and the overall free energy difference between the Fe–CH_2_CH_2_–S (**L**) intermediate and the products of −31.3 kcal mol^–1^.

**Figure 15:**
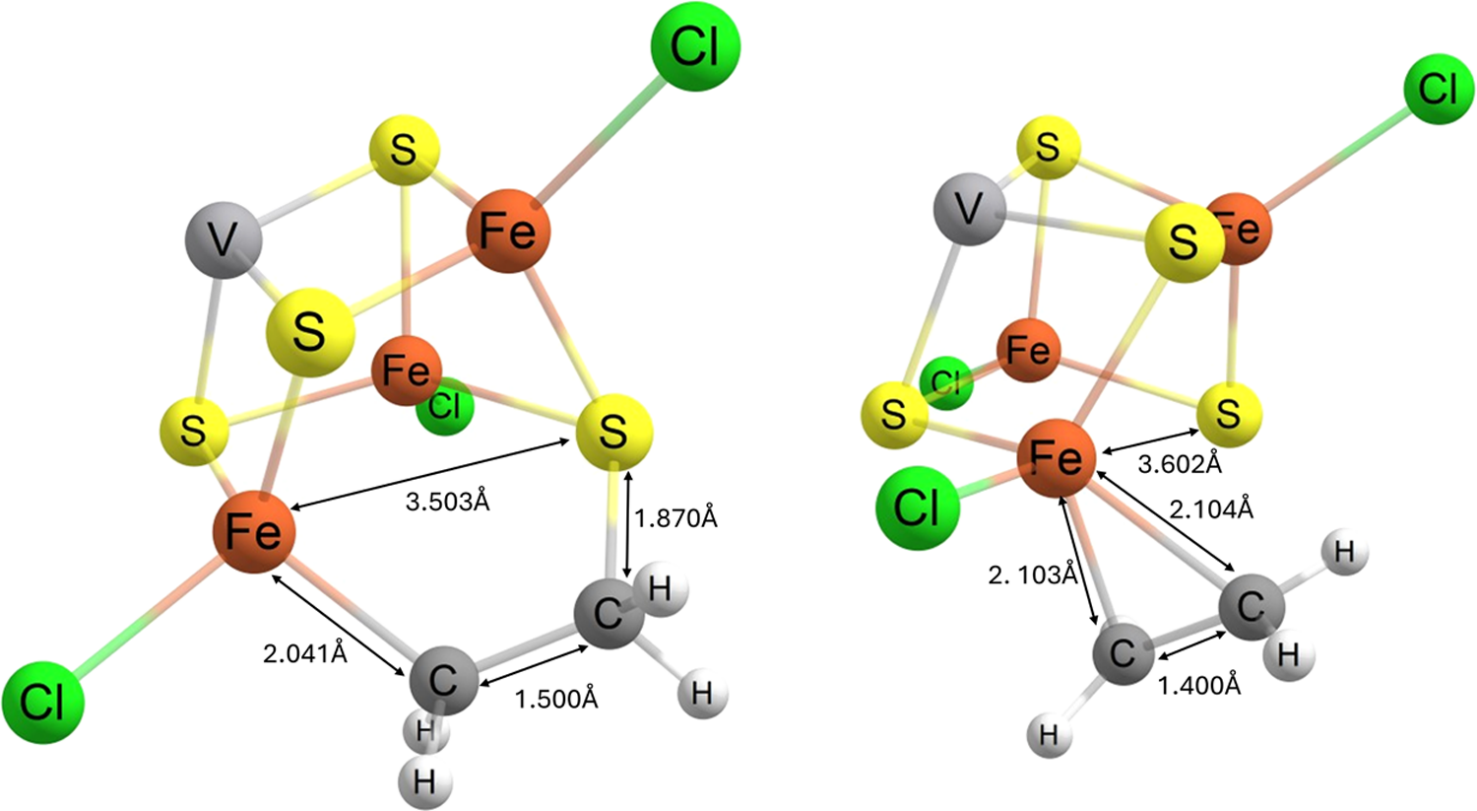
Structures of key CH_2_CH_2_-containing intermediates (**L**^–^ → **L**_bi_^–^ on Figure 16). Parts have been omitted for clarity.

**Figure 16:**
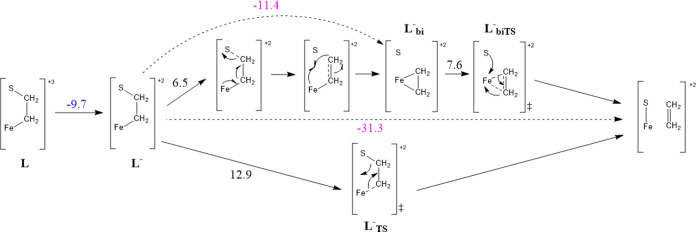
Summary of the mechanistic steps and energies involved in the dissociation of ethylene from the complex. Reduction free energies are given in blue, other free energies in pink, and electronic energies (from relaxed surface scans of bond lengths) in black. The relevant energies given are for structures with acetonitrile dissociated, and are given in kcal mol^–1^.

At the same time, an alternate, more stable configuration of the intermediate could be assumed via cleavage of the S–C bond (Figure 15). Scanning this bond shows a lesser barrier of 6.5 kcal mol^–1^ and formation of a terminal Fe–CH_2_CH_2_ species, which can then reorient and preferentially bind to the iron center in a sideways manner (**L**_bi_^–^). The Δ*G* between the Fe–CH_2_CH_2_–S (**L**^–^) and “sideways” Fe–CH_2_CH_2_ (**L**_bi_^–^) configurations is −11.4 kcal mol^–1^. From this intermediate, dissociation of ethylene can occur after a barrier of about Δ*E* = 7.6 kcal mol^–1^. The steps following the formation of the initial Fe–CH_2_CH_2_–S (**L**) intermediate up until the dissociation of ethylene are summarized in Figure 16.

Next to consider is the possibility of proceeding further toward a saturated product like ethane, another known product of CO’s reduction by nitrogenase enzymes. In this regard, we have considered 3 pathways—one starting from the Fe-COCH_2_ intermediate mentioned earlier in the pathway, one from the early stretching/dissociation of the S–C bond of the Fe–COHCH_2_–S (**H**) intermediate, and one from the aforementioned CH_2_–CH_2_–containing intermediates. In the latter case, protonation on a carbon center resulting in the formation of an Fe–CH_2_CH_3_ (**N**_sat_) species is a natural outcome following the “sideways” intermediate (**L**_bi_^–^). Considering starting from the Fe–CH_2_CH_2_–S (**L**) intermediate instead, scans indicate that the S–C bond is substantially weaker than the Fe–C bond at this stage, and so the Fe-CH_2_CH_3_ (**N**_sat_) intermediate is the more likely follow-up in this stage as well. The final step would then be another partial or full dissociation of the substrate for another protonation. As the product at this stage is a CH_2_CH_3_ radical, it is once again difficult to properly calculate a dissociation barrier, but from S-CH_2_CH_3_ it is +11.7 kcal mol^–1^ for a 0.5 Å stretch and a plateau of about 15 kcal mol^–1^, while from Fe-CH_2_CH_3_ (**N**_sat_) it is +9.6 kcal mol^–1^ for a 0.5 Å stretch and a plateau of about 18 kcal mol^–1^. As before, more detailed data from relaxed surface scans can be found in the Supporting Information.

**Figure 17:**
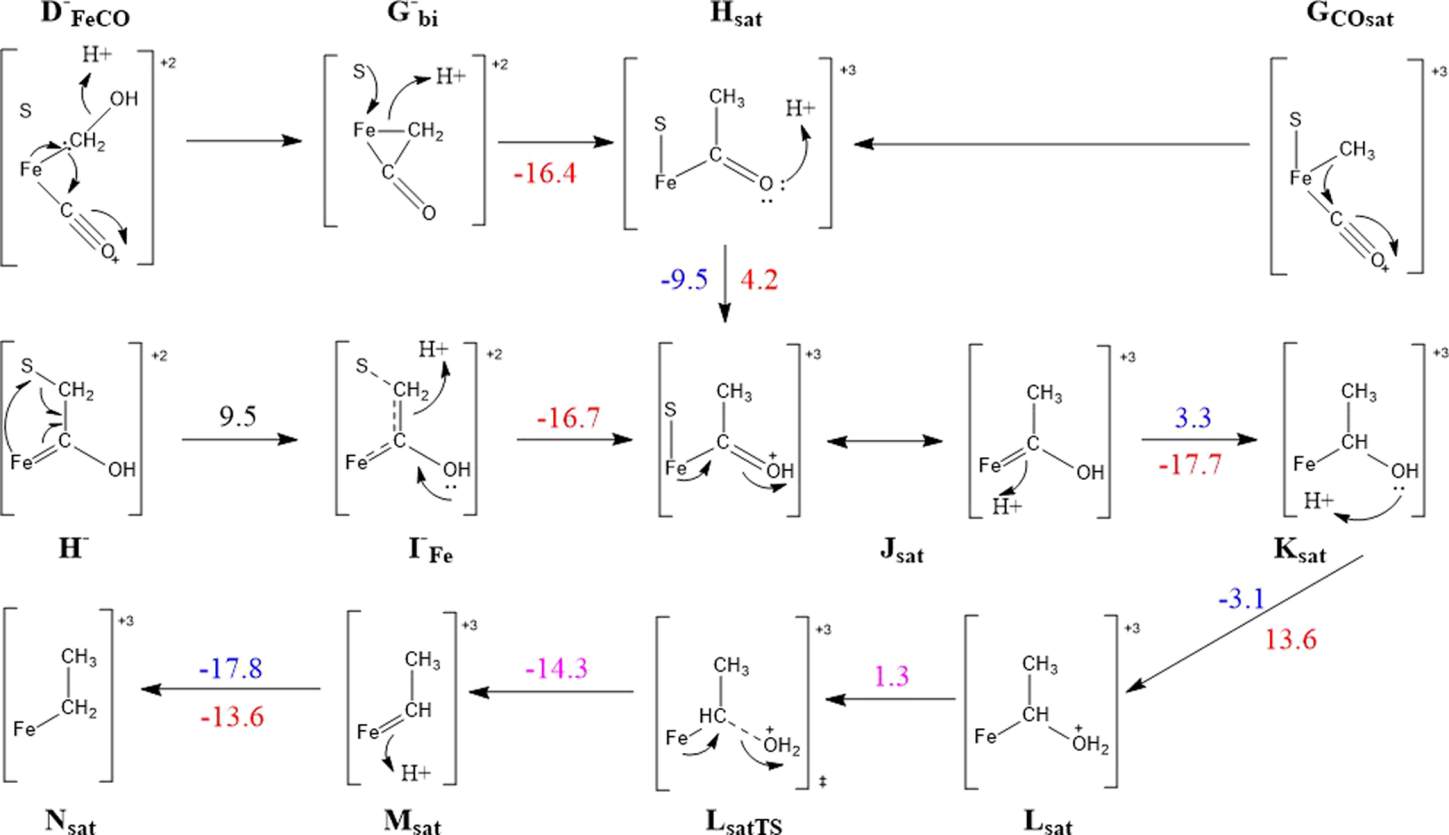
Summary of investigated mechanistic steps toward the formation of the C_2_H_6_ saturated product. Protonation free energies are given in red, reduction free energies in blue, other free energies in pink, and electronic energies (from relaxed surface scans) in black. When reduction and protonation energies are given together, reduction occurs first. The relevant energies given are for structures with acetonitrile dissociated, and are given in kcal mol^–1^.

Formation of a saturated product could be locked in earlier; we discussed earlier in this text the possibility of the formation of a Fe–COCH_2_ intermediate by means of concurrent C–C bond formation and H_2_O dissociation. While calculations suggest that such an intermediate would more favorably shift to the Fe–COCH_2_–S intermediate instead, if the S–CH_2_ bond is not formed (or not formed promptly enough, **D**_FeCO_^–^/**G**_bi_^–^), the −CH_2_ becomes by far the most favorable protonation site (exergonic by 16.4 kcal mol^–1^), resulting in the Fe–COCH_3_ (**H**_sat_) intermediate, which can also be produced through the C–C bond formation from the OC–Fe–CH_3_ (**G**_COsat_) intermediate. Further protonation and reduction steps from this intermediate have Δ*G* of −9.5 and 4.2 kcal mol^–1^ for reduction and protonation, respectively, toward the Fe–COH–CH_3_ (**J**_sat_) intermediate. The early cleavage of the S–CH_2_ bond of the Fe–C(OH)CH_2_–S (**H**^–^) species resulting in a Fe–C(OH)CH_2_ (**I**_Fe_^–^) intermediate has a scan-derived barrier Δ*E* ≈ 9.5 kcal mol^–1^. This is possible from this particular intermediate, as the breakage of the S–C bond allows the partial formation of the C–C double bond. The IBOs indicate the formation of the double bond in this case occurs through a simultaneous 1-electron donation from the β component of the S–C bond as well as the α component of the Fe–C π-bond. The β component localizes fully into a C–C π-bond while the α component remains strongly delocalized between the Fe–C–C centers. To avoid formation of an S radical, an electron from the Fe–S network replenishes the one lost from the S–C bond (in this particular case, an electron formerly delocalized between the Fe2 and Fe3 centers). Once this intermediate is formed, protonation of CH_2_ becomes the favored step, forming Fe–COHCH_3_ (**J**_sat_). The rest of the mechanism following this plays out in much the same way as from the Fe–COHCH_2_–S (**H**) intermediate, proceeding most favorably through the Fe–CH(OH)CH_3_ (**K**_sat_), Fe–CH(OH_2_)CH_3_ (**L**_sat_) and Fe-CHCH_3_ (**M**_sat_) intermediates before once again arriving at Fe–CH_2_CH_3_ (**N**_sat_). The steps and energies for this are summarized in Figure 17. Although potential products with 3 or more carbon atoms were not considered for this study, it would make the most sense for any subsequent C–C bond formations to occur in a manner similar to the first, such as from an S–CH_2_–CH–Fe (**K**) intermediate to S–CH_2_–CHCO–Fe.

Looking back at the proposed mechanisms as a whole, many of the intermediates calculated in this study suggest the formation and breakage of an iron–sulfur bond as something that can occur to either enable or stabilize the existence of certain intermediates. The spontaneous rearrangement of iron–sulfur cluster geometries as a result of changing electron configuration/redox occurring is something that has been documented previously for iron–sulfur clusters found in nature.^95,96^ The formation of carbon–sulfur bonds in relevant systems has also been previously proposed, although not a similar context.^97,98^ While we have found that binding of a substrate at any stage of the proposed mechanism between two Fe centers is not favorable compared to alternatives, the study of such a binding site on similar double-cubane-containing complexes^35^ may prove useful, as such an arrangement would more closely fit some of the proposals for the binding site of CO on the nitrogenase cofactors—in place of the relevant sulfur (Cl in model studied here).^50^ Such an arrangement could also potentially allow the CO-binding Fe center to adopt a configuration close to a tetrahedral high spin d^7^ as has been recently reported with the binding of CO to a [Fe_4_S_4_]^0^ cluster.^61^

#### 3.2.4 Binding on the Mo/V Centers

As binding of CO to the Mo/V centers in the absence of (or substituting) acetonitrile is plausible according to the calculated free energies, the first few steps of the mechanism were recalculated with such bindings in mind for the sake of completeness and comparison. Upon binding of the CO substrate to the Mo/V center, the lowest energy electronic configuration of the vanadium complex changes to Ms = 2, with the vanadium center formally adopting a similar non-Hund’s electronic structure to molybdenum, but with one α and one β electron. While this still retains the two V–Fe coupling connections as the two β variants, having one of each also allows it to maximize backbonding to the substrate. As molybdenum already has α and β for backbonding, it maintains an Ms = 1.5 configuration.

The free energies of the mechanism up to the M–CHOH (**C**) intermediate are given in Table 3, comparing the Mo/V binding sites between each other as well as the previously discussed Fe binding site on the vanadium complex. By comparing the lowest relative energy pathway between the starting point and the M–CHOH (**C**) intermediate, it is evident that the Fe binding site on the vanadium complex provides a mechanistic pathway with the smallest barriers, which is not surprising taking into account the activated carbon monoxide bond lengths as discussed in the Binding section. Molybdenum, due to its greater ability to store reducing equivalents as previously observed,^40^ remains only slightly inferior via the two consecutive reduction pathways (and not inferior if we were to consider acetonitrile remaining bound to V). The vanadium center as a binding and reduction site is remarkably worse, as the vanadium is unable to activate the substrate as well due to its smaller electron density compared to the iron and molybdenum, and this lack of activation is clear in the substantially larger initial protonation barrier.

**Table 3:** Δ*G* of Protonation/Reduction Steps of Carbon Monoxide When Bound to the Mo/V Centers of the [MoFe_3_S_4_]^3+^ and [VFe_3_S_4_]^3+^ Complexes as well as an Fe Center of the [VFe_3_S_4_]^3+^ Complex (with Acetonitrile Dissociated), Respectively

reaction Δ*G* with given complex/kcal mol^–1^			
step	Mo	V	V(Fe)
[Fe_3_S_4_M–CO]^3+^ → [Fe_3_S_4_M–CO]^2+^	–2.1	–14.8	–2.2
[Fe_3_S_4_M–CO]^2+^ → [Fe_3_S_4_M–CHO]^3+^	15.5	32.4	12.8
[Fe_3_S_4_M–CO]^2+^ → [Fe_3_S_4_M–CO]^1+^	9.7	17.8	12.9
[Fe_3_S_4_M–CHO]^3+^ → [Fe_3_S_4_M–CHO]^2+^	–14.3	–17.8	–7.4
[Fe_3_S_4_M–CO]^1+^ → [Fe_3_S_4_M–CHO]^2+^	–8.5	–3.2	–7.5
[Fe_3_S_4_M–CHO]^2+^ → [Fe_3_S_4_M–CHOH]^3+^	13.0	12.7	7.0

## 4 Conclusions

In conclusion, we have conducted a computational study on the intermediates of a hypothetical CO-reduction mechanism catalyzed by the biomimetic [MFe_3_S_4_]^3+^ cubanes that share some of the geometric and electronic structure features of the nitrogenase cofactors. We have proposed the order of most favorable intermediates and plausible mechanisms for how reductions to form CH_4_, C_2_H_4_, and C_2_H_6_ could proceed if catalyzed by these complexes, and investigated the features of the complexes which would drive the reactions. The results suggest that the tendency of molybdenum to delocalize its electrons more strongly to the iron centers, which was the major reason for its deemed greater efficacy of reducing hydrazine as was suggested in our last study, is likewise one of the major reasons for its lower efficacy with regard to reducing carbon monoxide, as the establishment of proper π-backbonding to the CO (as well as a number of other intermediates) is in competition with the Fe–Mo/V coupling. In the case of the studied model, this means that binding of the substrate to the Fe centers in the molybdenum complex is much less favorable than the equivalent of the vanadium complex, to the point where it would not occur under the conditions of the model and would require binding primarily on the Mo center, which would also prohibit formation of products with more than one carbon atom. This same effect with respect to the Mo/V–Fe coupling and backbonding could be part of the reason for the discrepancy between the molybdenum and vanadium nitrogenases’ efficacy at carbon monoxide reduction. In general, many of the calculated intermediates make use of the remarkable flexibility of the iron–sulfur cluster in stabilizing a wide range of intermediates through partial/1-electron bonds as well as a variable arrangement of bonding between the iron and sulfur centers. In addition, the extensive electron delocalization, as observed between the Fe–Fe centers as with the mixed-valence electron, and between the Mo/V–Fe centers as antiferromagnetic coupling, serves to both stabilize intermediates and drive the reactions forward through “back-and-forth” action. Oxygen-containing products (other than the water byproduct) are avoided due to carbon maintaining preferential binding to the complex in the intermediates, which is consistent with the literature of nitrogenases. The relative intermediates and scans’ energies also explain the naturally observed abundance of the C_2_H_4_ product as opposed to all others—both the formation of intermediates which lead to other products as well as the dissociation of the products themselves at the end of the catalytic cycle requires greater barriers than that of C_2_H_4_. The labile acetonitrile ligand on the Mo/V center can associate or dissociate to act as an additional stabilizing feature for the intermediates; this function of acetonitrile in this model could be in part duplicated by the histidine H442 of the nitrogenases.

## Supporting Information Available

Cartesian coordinates of optimized structures (ZIP)
Relaxed surface scan graphs; additional IBO figures; and energies and charge/spin populations of discussed intermediates (PDF)
