# Supplementary material for: Biomimetic [MFe3S4]3+ Cubanes (M = V/Mo) as Catalysts for a Fischer–Tropsch-like Hydrocarbon Synthesis—A Computational Study
Source: Inorg Chem. 2024 Dec 27;64(1):479–94. doi: 10.1021/acs.inorgchem.4c04995 (PMC11734119; doi:10.1021/acs.inorgchem.4c04995)
Supplement: Supplementary file 2 — ic4c04995_si_002.pdf [file ic4c04995_si_002.pdf]

Supporting Information

Biomimetic $[MFe_3S_4]^{3+}$ cubanes (M = V/Mo) as catalysts for a Fischer-Tropsch-like hydrocarbon synthesis – a computational study.

Maxim Barchenko,[†, ‡] Thomas Malcomson,[†] Patrick J. O'Malley,[†] and Sam P. de Visser*[,†,¶]

†Department of Chemistry, School of Natural Sciences, The University of Manchester, Oxford Road, Manchester, M13 9PL, UK.

‡Manchester Institute of Biotechnology, The University of Manchester, 131 Princess Street, Manchester M1 7DN, UK.

¶Department of Chemical Engineering, The University of Manchester, Oxford Road, Manchester, M13 9PL, UK.

E-mail: sam.devisser@manchester.ac.uk

Methods

Unless explicitly stated otherwise, all data presented in this document follows the methods as described in the main text. Relaxed surface scan graphs and IBOs use geometries and orbitals as obtained with the BP86 functional after optimization. The tables report energies and population analyses as obtained via the TPSSh single point calculations following optimization, with free energies calculated from the BP86 post-optimization frequency analysis. The charge of any given intermediate refers to its core charge, not overall charge. For example, starting complex $[VFe_3S_4]^{3+}$ has a core charge of +3 and overall charge of -2.

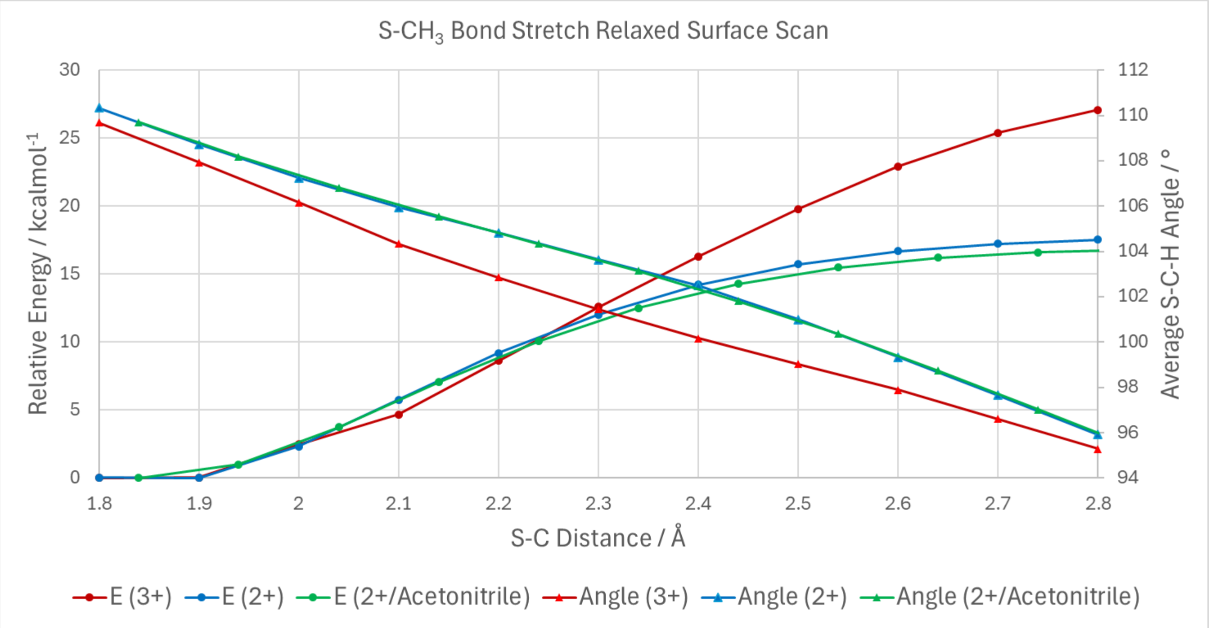

Figure S1 – Relaxed surface scans for the stretching of the S-C bond of the $S\text{-}CH_3$ intermediates.

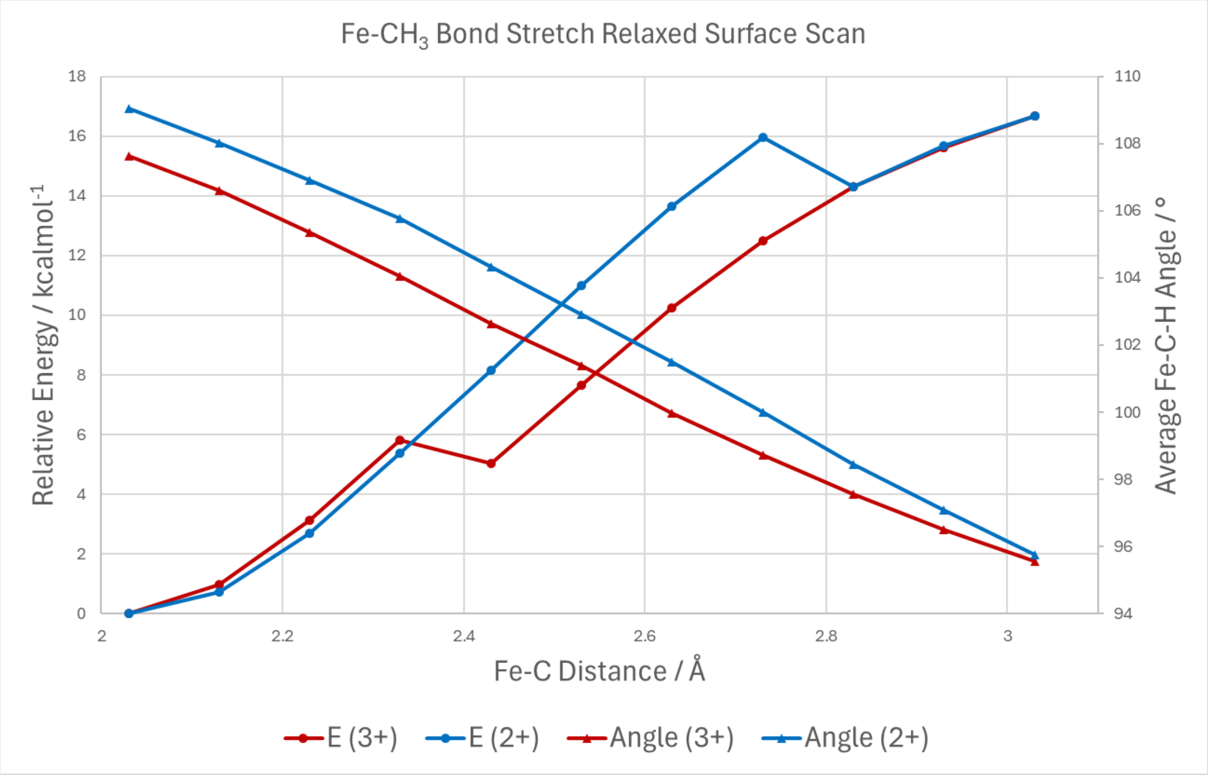

Figure S2 – Relaxed surface scans for the stretching of the Fe-C bond of the Fe-CH_3 intermediates.

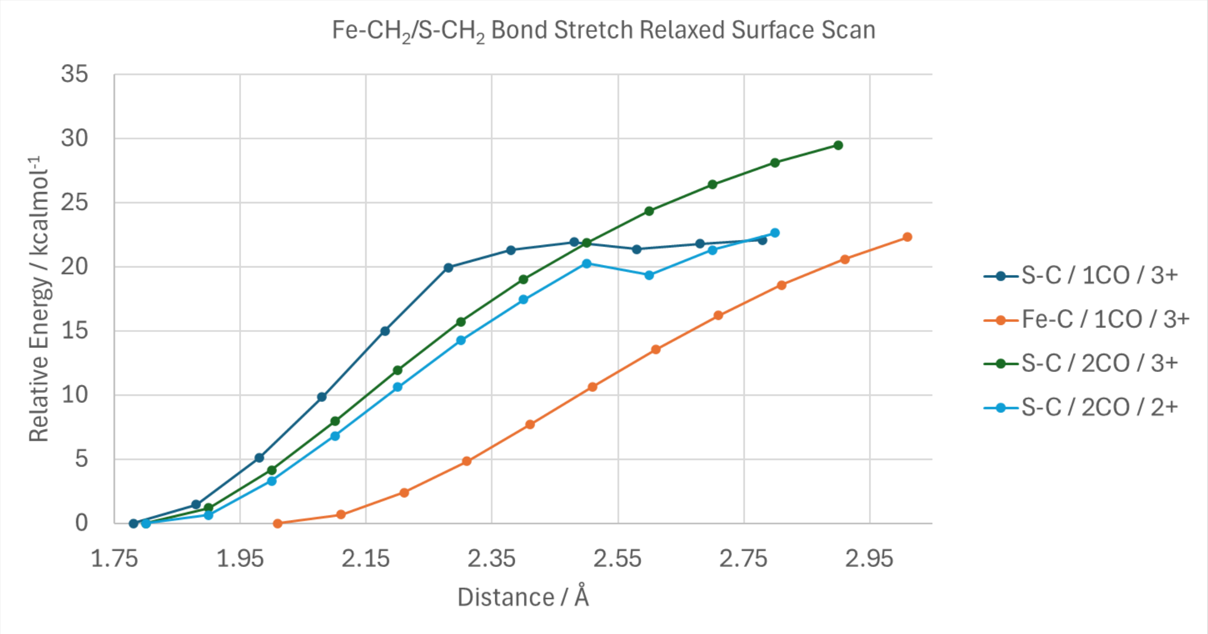

Figure S3 – Relaxed surface scans for the stretching of the Fe-C/S-C bonds from the Fe-CH_2-S and OC-Fe-CH_2-S intermediates.

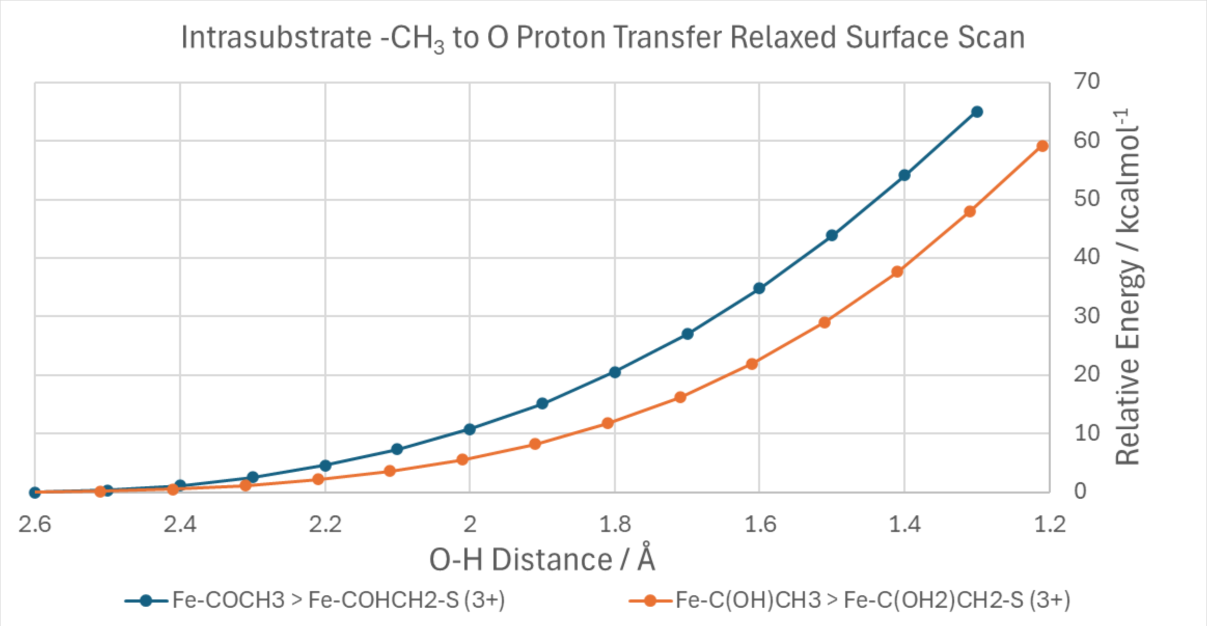

Figure S4 – Relaxed surface scans for the proton transfer between the methyl and carbonyl/alcohol groups of the Fe-$COCH_3$ (3+) and Fe-C(OH)CH_3 (3+) intermediates.

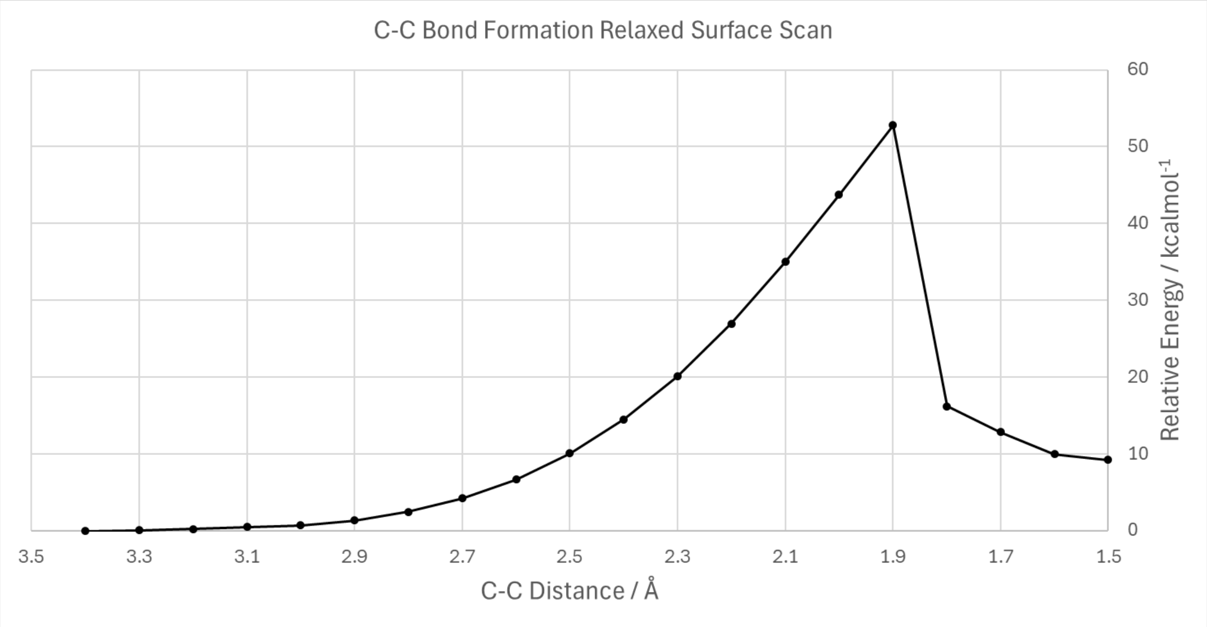

Figure S5 – Relaxed surface scan showing C-C bond formation from the Fe-CO/S-CH_3 intermediate.

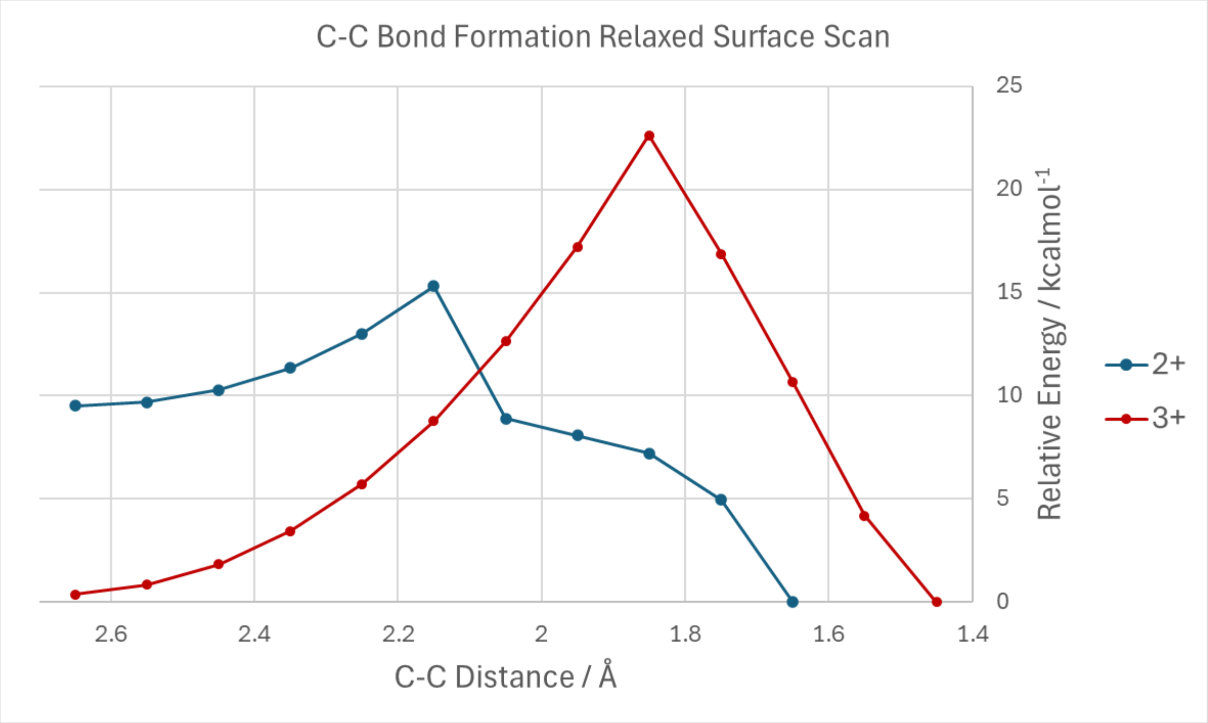

Figure S6 – Relaxed surface scan showing C-C bond formation from the OC-Fe-CH_2OH_2 intermediates with acetonitrile bound to the vanadium. We note similar barrier for H_2O dissociation in the 2+ species compared to the version without acetonitrile as well as a lack of second saddle for C-C bond formation itself. As discussed in the main text, the scans originating from the OC-Fe-CH_2OH_2 intermediates were performed with the Fe-Cl bond constrained.

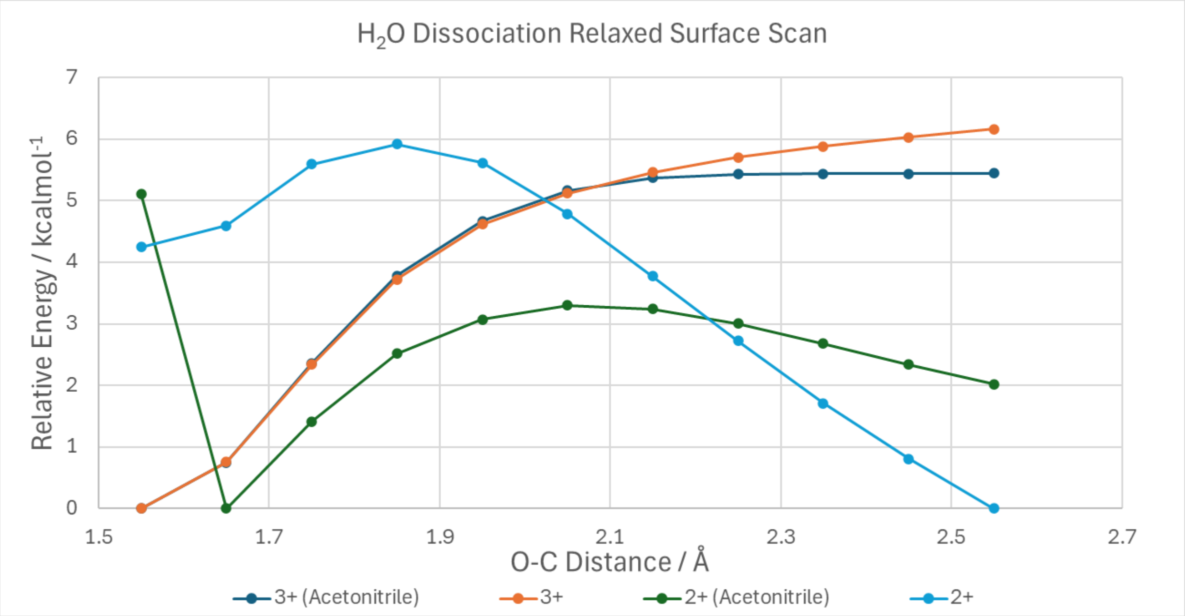

Figure S7 – Relaxed surface scans showing H_2O dissociation from the OC-Fe-CH_2OH_2 intermediates with and without acetonitrile bound to the vanadium. We note asymptote-like profile for the 3+ intermediates as there aren't enough electrons to provide to the H_2O while also stabilizing the substrate intermediate. We also note the long-range effect of acetonitrile of increasing the resting O-C bond distance due to the extra electron density it provides to the

vanadium, and by extension to the Fe-S cluster. As discussed in the main text, the scans originating from the OC-Fe-CH_2OH_2 intermediates were performed with the Fe-Cl bond constrained.

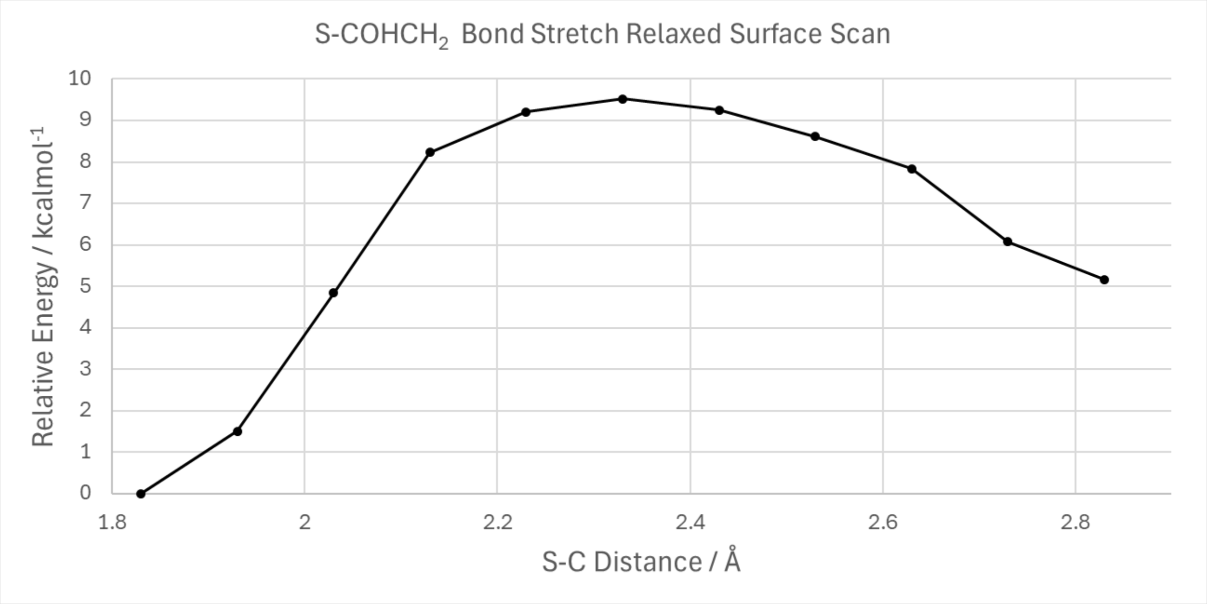

Figure S8 – Relaxed surface scan showing the stretching of the S-C bond of the Fe-C(OH)CH_2-S intermediate.

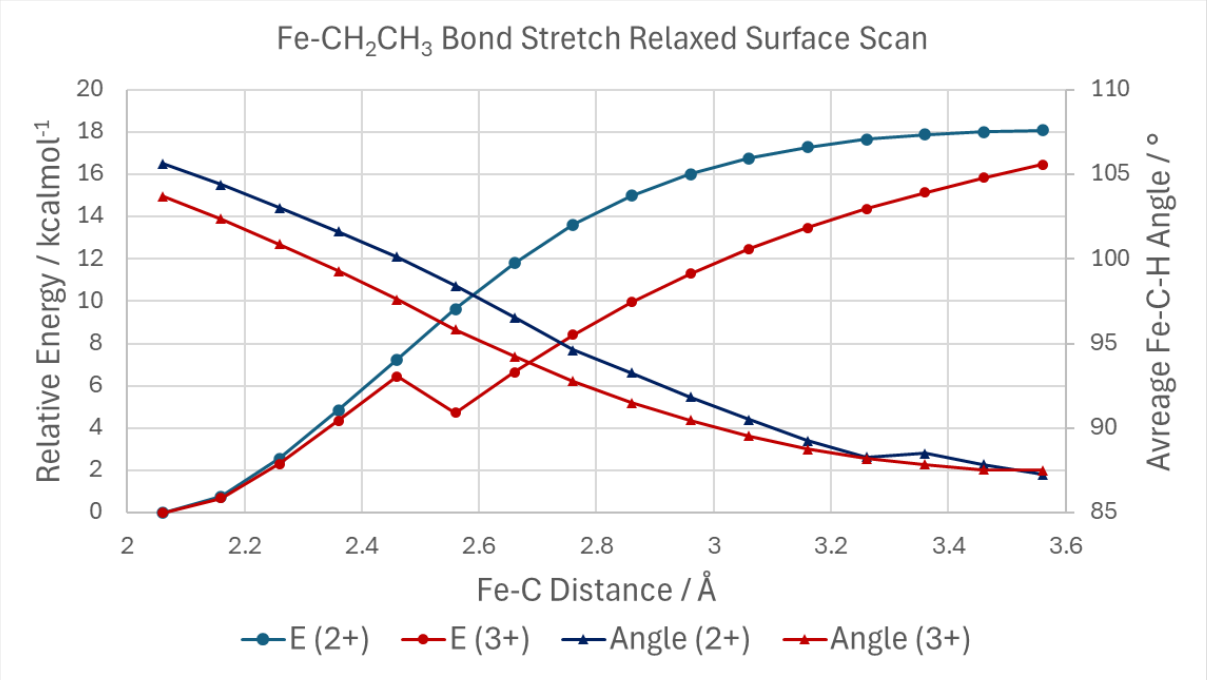

Figure S9 – Relaxed surface scans showing the stretching of the Fe-C bond of the Fe-CH_2CH_3 intermediate.

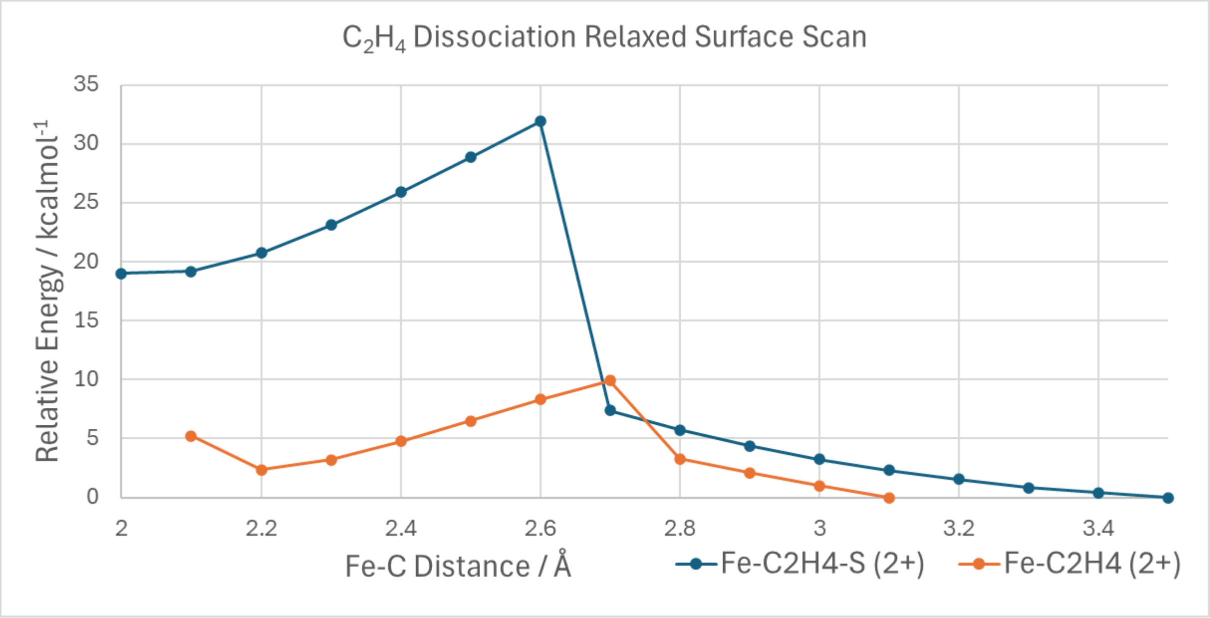

Figure S10 – Relaxed surface scans showing the dissociation of ethylene from the Fe-C_2H_4-S and Fe-C_2H_4 (side-on) intermediates.

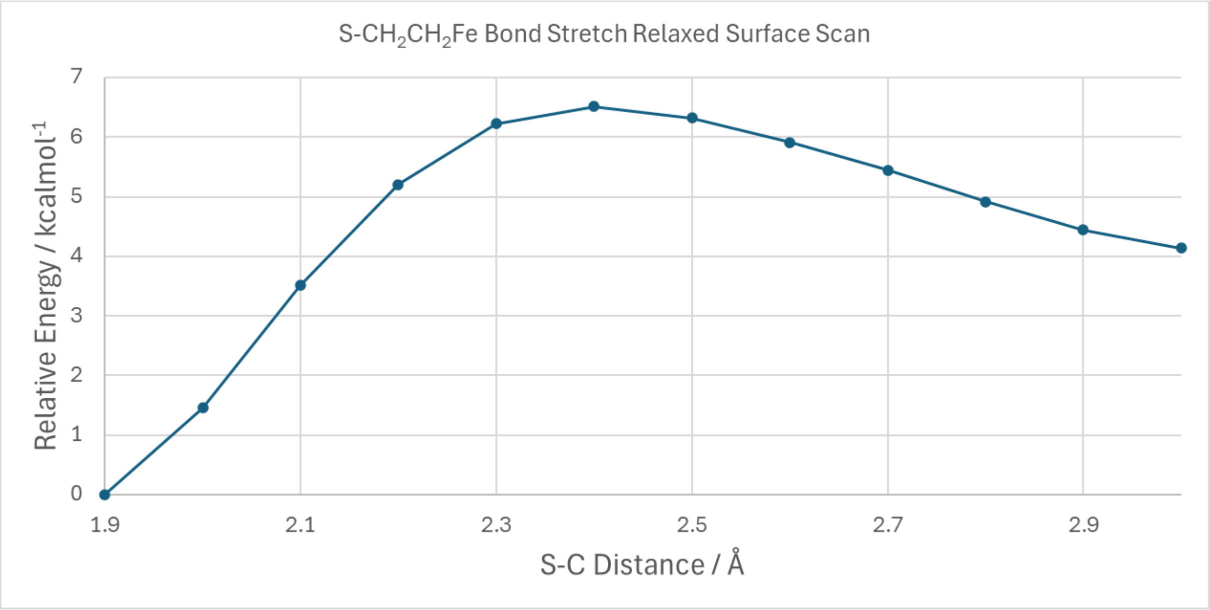

Figure S11 – Relaxed surface scan showing the stretching of the S-C bond from the S-CH_2CH_2-Fe intermediate.

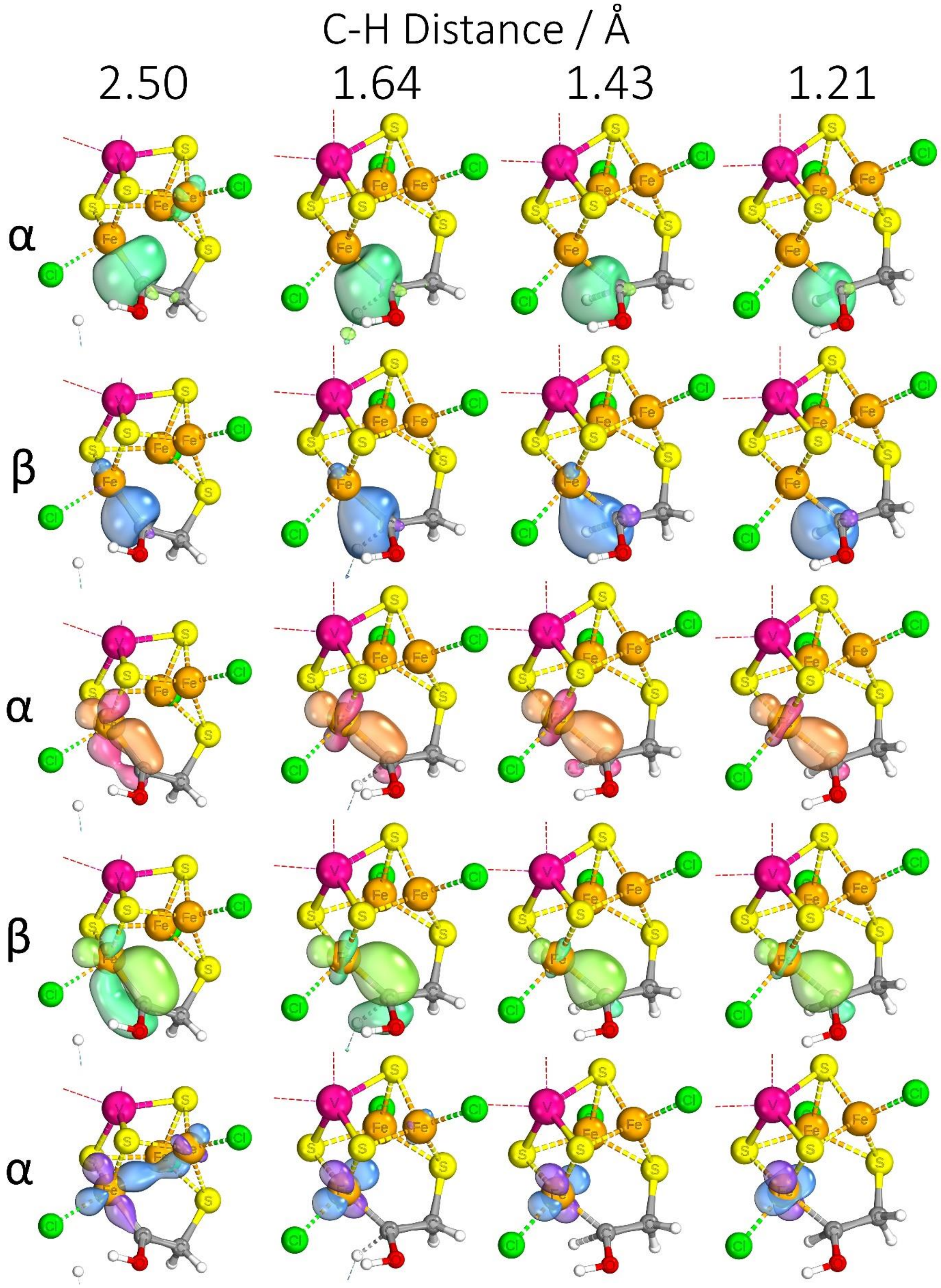

Figure S12 – IBOs from the relaxed surface scan for the protonation of the carbon centre in the Fe-C(OH)CH_2-S intermediate.

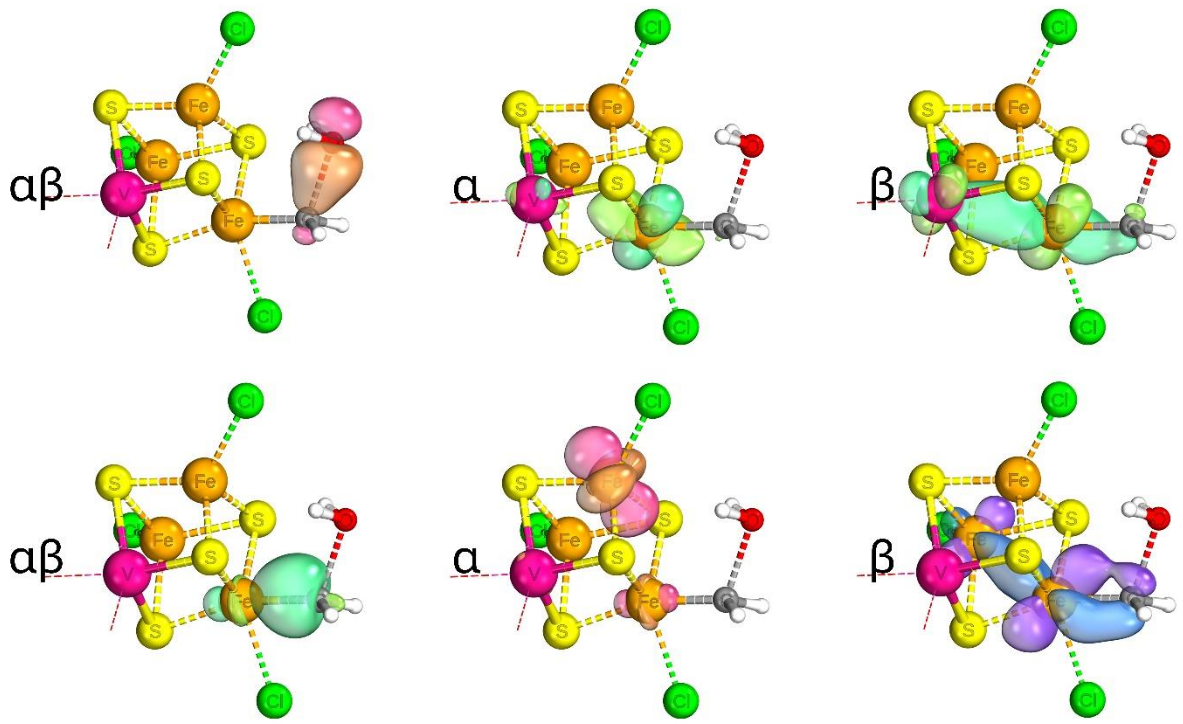

Figure S13 – IBOs from the optimized transition state of H_2O dissociation from the Fe-CH_2OH_2 (2+) intermediate, showing electron delocalization and flow to deliver reducing equivalents.

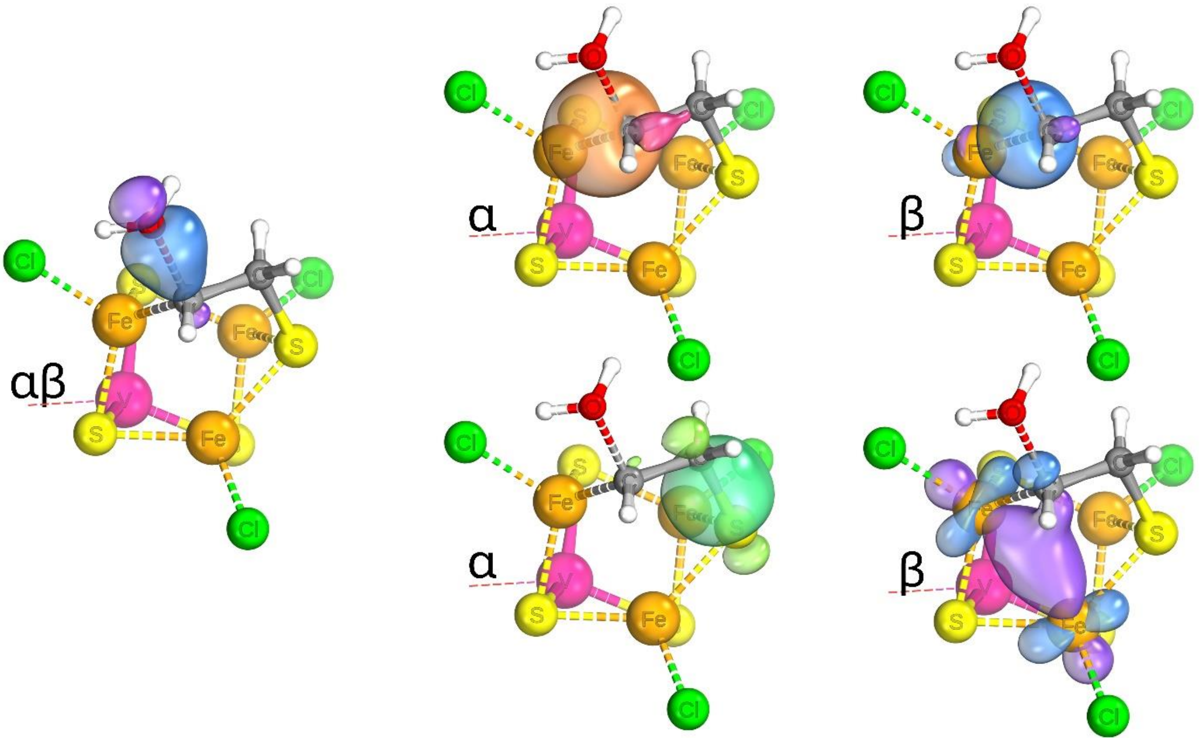

Figure S14 – IBOs from the optimized transition state of H_2O dissociation from the Fe-$CHOH_2CH_2$-S (3+) intermediate, showing electron delocalization and flow to deliver reducing equivalents.

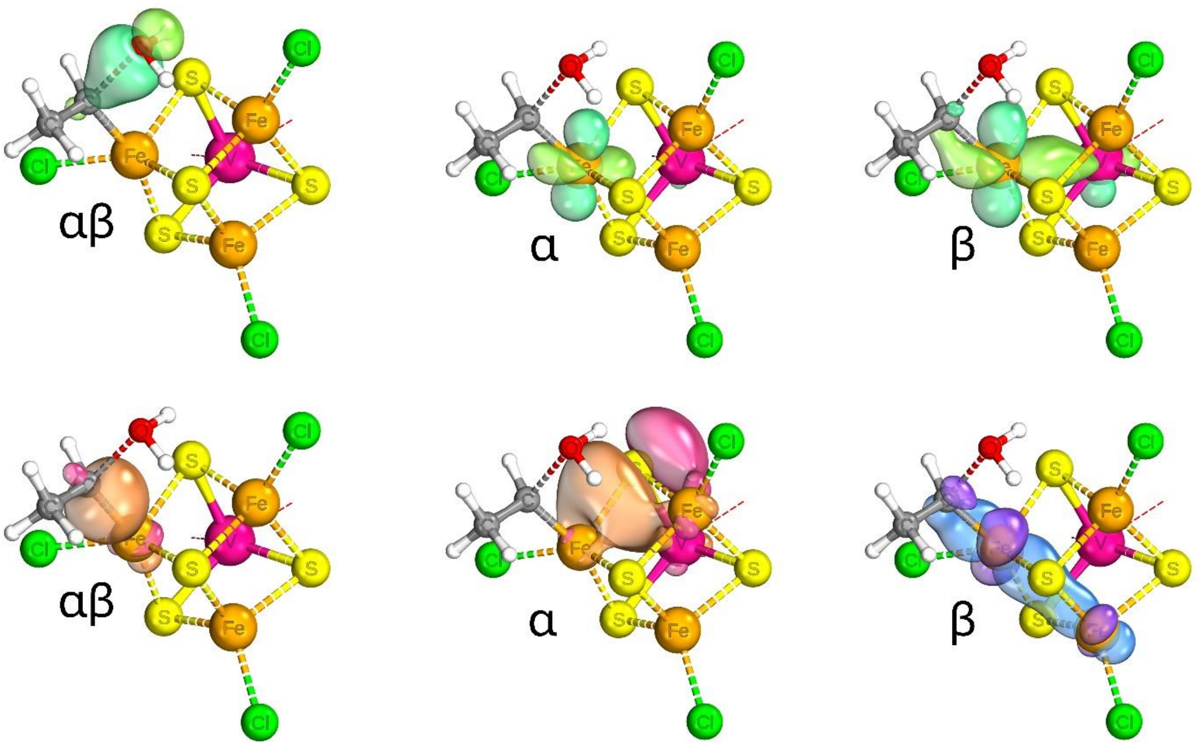

Figure S15 – IBOs from the optimized transition state of H_2O dissociation from the Fe-$CHOH_2CH_3$ (3+) intermediate, showing electron delocalization and flow to deliver reducing equivalents.

Table S1 – Electronic, Zero Point (ZPE), and Free Energies (in au) calculated for the optimized geometries of the vanadium complexes up to and including release of methane as a product.

Intermediate	E	ZPE	G
^{3}[Initial/ACN]$^{3+}$	-10062.893215	0.108585	-10062.862366
^{4}[Initial/ACN]$^{2+}$	-10063.060521	0.109279	-10063.014625
^{3}[Fe-CO/ACN]$^{3+}$	-10176.296374	0.118346	-10176.242931
^{2}[Fe-CO/ACN]$^{2+}$	-10176.424568	0.116367	-10176.375952
^{4}[Fe-CHO/ACN]$^{3+}$	-10176.863273	0.125914	-10176.804632
^{3}[Fe-CHO/ACN]$^{2+}$	-10176.998557	0.126322	-10176.480575
^{3}[Fe-CHOH/ACN]$^{3+}$	-10177.452493	0.141256	-10177.375160
^{3}[Fe-CO]$^{3+}$	-10043.463692	0.072657	-10043.452201
^{2}[Fe-CO]$^{2+}$	-10043.585615	0.071360	-10043.577995
^{4}[Fe-CHO]$^{3+}$	-10044.030231	0.080929	-10044.011614
^{3}[Fe-CHO]$^{2+}$	-10044.164586	0.081345	-10044.144937
^{3}[Fe-CHOH]$^{3+}$	-10044.620630	0.094166	-10044.587747
^{4}[Fe-CHOH]$^{2+}$	-10044.742876	0.092371	-10044.712665
^{2}[Fe-CH_2OH]$^{3+}$	-10045.223510	0.103838	-10045.181248
^{3}[Fe-CH_2OH]$^{2+}$	-10045.362405	0.103851	-10045.320580
^{3}[Fe-CH_2OH_2]$^{3+}$	-10045.806607	0.115405	-10045.752878
^{3}[Fe-CH_2-S]$^{3+}$	-9969.360342	0.089949	-9969.330509
^{4}[Fe-CH_2OH_2]$^{2+}$	-10045.921346	0.112469	-10045.871581
^{4}[Fe-CH_2-S]$^{2+}$	-9969.494836	0.088411	-9969.467241

$^{1}[Fe{=}CH_2]^{3+}$	-9969.310997	0.088236	-9969.282304
$^{2}[Fe{=}CH_2]^{2+}$	-9969.449443	0.086299	-9969.423501
$^{4}[Fe\text{-}CH_3]^{3+}$	-9969.954916	0.099865	-9969.916720
$^{3}[Fe\text{-}CH_3]^{2+}$	-9970.078320	0.098653	-9970.041419
$^{4}[S\text{-}CH_3]^{3+}$	-9969.999201	0.100044	-9969.961871
$^{3}[S\text{-}CH_3]^{2+}$	-9970.125213	0.098916	-9970.088553
$^{4}[\text{Initial}]^{2+}$	-9930.223635	0.064179	-9930.219084

Table S2 – Mulliken spin population analyses of the transition metals and substrate C/O atoms in the optimized geometries of the vanadium complexes up to and including release of methane as a product.

Intermediate	V	Fe1	Fe2	Fe3	C	O
$^{3}[\text{Initial/ACN}]^{3+}$	-1.71	3.48	3.54	-3.65	N/A	N/A
$^{4}[\text{Initial/ACN}]^{2+}$	-1.42	3.51	3.51	-3.40	N/A	N/A
$^{3}[Fe\text{-}CO/ACN]^{3+}$	0.22	3.55	1.92	-3.54	-0.12	-0.04
$^{2}[Fe\text{-}CO/ACN]^{2+}$	-1.52	3.60	1.94	-3.38	-0.13	-0.05
$^{4}[Fe\text{-}CHO/ACN]^{3+}$	0.03	3.53	2.93	-3.53	-0.16	-0.08
$^{3}[Fe\text{-}CHO/ACN]^{2+}$	-1.22	3.55	2.74	-3.40	-0.16	-0.08
$^{3}[Fe\text{-}CHOH/ACN]^{3+}$	0.04	3.55	2.05	-3.49	-0.24	-0.03
$^{3}[Fe\text{-}CO]^{3+}$	0.58	3.46	1.88	-3.56	-0.09	-0.03
$^{2}[Fe\text{-}CO]^{2+}$	0.80	3.44	0.51	-3.47	-0.03	0.00
$^{4}[Fe\text{-}CHO]^{3+}$	0.41	3.48	2.97	-3.56	-0.19	-0.12
$^{3}[Fe\text{-}CHO]^{2+}$	-0.38	3.44	2.55	-3.49	-0.17	-0.09
$^{3}[Fe\text{-}CHOH]^{3+}$	0.51	3.46	2.01	-3.53	-0.22	-0.03
$^{4}[Fe\text{-}CHOH]^{2+}$	-0.56	3.28	2.05	-3.41	-0.31	-0.04
$^{2}[Fe\text{-}CH_2OH]^{3+}$	-1.51	3.52	2.93	-3.73	-0.29	-0.06
$^{3}[Fe\text{-}CH_2OH]^{2+}$	-0.74	3.45	2.68	-3.45	-0.26	-0.04
$^{3}[Fe\text{-}CH_2OH_2]^{3+}$	-0.02	3.46	2.21	-3.52	-0.13	0.00
$^{3}[Fe\text{-}CH_2\text{-}S]^{3+}$	-0.51	3.39	2.79	-3.49	-0.31	N/A
$^{4}[Fe\text{-}CH_2OH_2]^{2+}$	0.47	3.41	2.31	-3.25	-0.13	0.00
$^{4}[Fe\text{-}CH_2\text{-}S]^{2+}$	-0.92	3.30	3.57	-3.43	0.10	N/A
$^{1}[Fe{=}CH_2]^{3+}$	0.68	3.43	-0.62	-3.53	0.44	N/A
$^{2}[Fe{=}CH_2]^{2+}$	-0.73	3.47	2.28	-3.44	-0.88	N/A
$^{4}[Fe\text{-}CH_3]^{3+}$	-1.11	3.38	2.74	-2.86	0.28	N/A
$^{3}[Fe\text{-}CH_3]^{2+}$	-1.41	3.36	2.20	-2.83	0.25	N/A
$^{4}[S\text{-}CH_3]^{3+}$	-1.09	3.50	3.43	-3.44	0.00	N/A
$^{3}[S\text{-}CH_3]^{2+}$	-1.40	3.31	3.21	-3.46	-0.02	N/A
$^{4}[\text{Initial}]^{2+}$	-0.90	3.43	3.43	-3.45	N/A	N/A

Table S3 – Mulliken atomic charge analyses of the transition metals and substrate C/O atoms in the optimized geometries of the vanadium complexes up to and including release of methane as a product.

Intermediate	V	Fe1	Fe2	Fe3	C	O
$^{3}[\text{Initial/ACN}]^{3+}$	0.43	0.49	0.52	0.52	N/A	N/A
$^{4}[\text{Initial/ACN}]^{2+}$	0.42	0.50	0.50	0.51	N/A	N/A
$^{3}[Fe\text{-}CO/ACN]^{3+}$	0.42	0.51	0.24	0.51	0.22	-0.15
$^{2}[Fe\text{-}CO/ACN]^{2+}$	0.44	0.52	0.26	0.50	0.18	-0.19

^{4}[Fe-CHO/ACN]$^{3+}$	0.40	0.51	0.24	0.52	0.21	-0.27
^{3}[Fe-CHO/ACN]$^{2+}$	0.44	0.51	0.22	0.51	0.20	-0.33
^{3}[Fe-CHOH/ACN]$^{3+}$	0.41	0.52	0.16	0.42	0.15	-0.27
^{3}[Fe-CO]$^{3+}$	0.64	0.53	0.17	0.53	0.28	-0.12
^{2}[Fe-CO]$^{2+}$	0.61	0.52	0.19	0.50	0.21	-0.19
^{4}[Fe-CHO]$^{3+}$	0.64	0.53	0.23	0.53	0.22	-0.25
^{3}[Fe-CHO]$^{2+}$	0.61	0.50	0.21	0.53	0.19	-0.34
^{3}[Fe-CHOH]$^{3+}$	0.64	0.52	0.16	0.43	0.16	-0.26
^{4}[Fe-CHOH]$^{2+}$	0.61	0.50	0.21	0.43	0.09	-0.31
^{2}[Fe-CH$_2$OH]$^{3+}$	0.68	0.53	0.23	0.56	0.01	-0.41
^{3}[Fe-CH$_2$OH]$^{2+}$	0.61	0.50	0.26	0.49	-0.02	-0.45
^{3}[Fe-CH$_2$OH$_2$]$^{3+}$	0.60	0.52	0.24	0.42	-0.17	-0.27
^{3}[Fe-CH$_2$-S]$^{3+}$	0.56	0.45	0.42	0.48	-0.19	N/A
^{4}[Fe-CH$_2$OH$_2$]$^{2+}$	0.55	0.50	0.29	0.42	-0.20	-0.26
^{4}[Fe-CH$_2$-S]$^{2+}$	0.54	0.46	0.53	0.47	-0.40	N/A
^{1}[Fe=CH$_2$]$^{3+}$	0.67	0.52	0.24	0.53	-0.18	N/A
^{2}[Fe=CH$_2$]$^{2+}$	0.62	0.52	0.37	0.51	-0.36	N/A
^{4}[Fe-CH$_3$]$^{3+}$	0.64	0.50	0.31	0.48	-0.38	N/A
^{3}[Fe-CH$_3$]$^{2+}$	0.62	0.49	0.35	0.47	-0.41	N/A
^{4}[S-CH$_3$]$^{3+}$	0.60	0.48	0.53	0.49	-0.34	N/A
^{3}[S-CH$_3$]$^{2+}$	0.56	0.46	0.49	0.49	-0.35	N/A
^{4}[Initial]$^{2+}$	0.61	0.49	0.49	0.52	N/A	N/A

Table S4 – Electronic, Zero Point (ZPE), and Free Energies (in au) calculated for the optimized geometries of the vanadium complexes involving the binding of 2CO substrates, up to but not including C-C bond formation. * - structure optimized with a Fe-Cl bond constraint.

Intermediate	E	ZPE	G
^{1}[OC-Fe-CO/ACN]$^{3+}$	-10289.693910	0.125529	-10289.634413
^{8}[OC-Fe-CO/ACN]$^{2+}$	-10289.821271	0.123520	-10289.768053
^{2}[OC-Fe-CHO/ACN]$^{3+}$	-10290.248937	0.134973	-10290.182312
^{1}[OC-Fe-CHO/ACN]$^{2+}$	-10290.385547	0.134065	-10290.318092
^{1}[OC-Fe-CHOH/ACN]$^{3+}$	-10290.840036	0.146619	-10290.761418
^{2}[OC-Fe-CHOH/ACN]$^{2+}$	-10290.955784	0.144177	-10290.880614
^{2}[OC-Fe-CH$_2$OH/ACN]$^{3+}$	-10291.451342	0.157617	-10291.362962
^{1}[OC-Fe-CH$_2$OH/ACN]$^{2+}$	-10291.577570	0.156872	-10291.488699
^{1}[OC-Fe-CH$_2$OH]$^{2+}$	-10158.748710	0.111910	-10158.700000
^{1}[OC-Fe-CH$_2$OH$_2$]$^{3+}$*	-10159.196378	0.123244	-10082.671193
^{2}[OC-Fe-CH$_2$OH$_2$]$^{2+}$*	-10159.301260	0.120671	-10082.801070
^{1}[OC-Fe=CH$_2$]$^{3+}$*	-10082.704277	0.095283	-10082.671193
^{2}[OC-Fe=CH$_2$]$^{2+}$	-10082.831279	0.093164	-10082.801069
^{3}[OC-Fe-CH$_2$-S]$^{3+}$	-10082.739308	0.096470	-10082.705717
^{2}[OC-Fe-CH$_2$-S]$^{2+}$	-10082.877386	0.095994	-10082.844299
^{2}[OC-Fe-CH$_3$]$^{3+}$	-10083.361742	0.107076	-10083.317179
^{1}[OC-Fe-CH$_3$]$^{2+}$	-10083.498558	0.106850	-10083.453849

Table S5 – Mulliken spin population analyses of the transition metals and substrate C/O atoms in the optimized geometries of the vanadium complexes involving the binding of 2CO substrates, up to but not including C-C bond formation. * - structure optimized with a Fe-Cl bond constraint.

Intermediate	V	Fe1	Fe2	Fe3	C	O	C	O
^{1}[OC-Fe-CO/ACN]$^{3+}$	0.13	3.53	-0.01	-3.56	0.01	0.00	-0.01	0.00
^{8}[OC-Fe-CO/ACN]$^{2+}$	-1.58	3.57	0.07	3.57	-0.01	0.00	0.00	0.00
^{2}[OC-Fe-CHO/ACN]$^{3+}$	0.14	3.53	1.16	-3.52	-0.07	-0.03	0.06	-0.02
^{1}[OC-Fe-CHO/ACN]$^{2+}$	0.11	3.53	-0.04	-3.52	-0.01	0.00	0.01	0.00
^{1}[OC-Fe-CHOH/ACN]$^{3+}$	0.10	3.53	-0.04	-3.54	0.00	0.00	0.01	0.00
^{2}[OC-Fe-CHOH/ACN]$^{2+}$	1.46	3.41	0.01	-3.54	0.01	0.00	0.01	0.00
^{2}[OC-Fe-CH$_2$OH/ACN]$^{3+}$	0.09	3.53	1.25	-3.53	-0.13	-0.02	-0.06	-0.02
^{1}[OC-Fe-CH$_2$OH/ACN]$^{2+}$	0.18	3.53	-0.12	-3.51	-0.03	0.00	0.01	0.00
^{1}[OC-Fe-CH$_2$OH]$^{2+}$	-0.05	3.46	0.05	-3.48	0.03	0.00	-0.01	0.00
^{1}[OC-Fe-CH$_2$OH$_2$]$^{3+}$*	0.51	3.43	0.02	-3.59	0.00	0.00	0.00	0.00
^{2}[OC-Fe-CH$_2$OH$_2$]$^{2+}$*	0.26	3.41	1.36	-3.54	-0.05	-0.01	-0.09	-0.04
^{1}[OC-Fe=CH$_2$]$^{3+}$*	0.07	3.48	-0.14	-3.49	0.12	N/A	0.00	0.00
^{2}[OC-Fe=CH$_2$]$^{2+}$	-0.04	3.46	0.15	-3.47	1.02	N/A	-0.04	-0.01
^{3}[OC-Fe-CH$_2$-S]$^{3+}$	0.03	3.49	2.13	-3.49	-0.06	N/A	-0.11	-0.03
^{2}[OC-Fe-CH$_2$-S]$^{2+}$	-0.91	3.37	2.13	-3.46	-0.08	N/A	-0.12	-0.04
^{2}[OC-Fe-CH$_3$]$^{3+}$	-0.54	3.58	1.17	-3.44	-0.04	N/A	-0.07	-0.02
^{1}[OC-Fe-CH$_3$]$^{2+}$	-0.65	3.55	0.14	-3.43	0.03	N/A	-0.01	0.00

Table S6 – Mulliken atomic charge analyses of the transition metals and substrate C/O atoms in the optimized geometries of the vanadium complexes involving the binding of 2CO substrates, up to but not including C-C bond formation. * - structure optimized with a Fe-Cl bond constraint.

Intermediate	V	Fe1	Fe2	Fe3	C	O	C	O
^{1}[OC-Fe-CO/ACN]$^{3+}$	0.34	0.49	0.11	0.50	0.23	-0.12	0.22	-0.12
^{8}[OC-Fe-CO/ACN]$^{2+}$	0.32	0.49	0.18	0.50	0.21	-0.16	0.20	-0.16
^{2}[OC-Fe-CHO/ACN]$^{3+}$	0.32	0.51	0.26	0.51	0.17	-0.28	0.12	-0.13
^{1}[OC-Fe-CHO/ACN]$^{2+}$	0.35	0.50	0.10	0.49	0.15	-0.36	0.18	-0.20
^{1}[OC-Fe-CHOH/ACN]$^{3+}$	0.36	0.50	0.13	0.50	0.07	-0.31	0.24	-0.15
^{2}[OC-Fe-CHOH/ACN]$^{2+}$	0.40	0.49	0.24	0.48	0.03	-0.34	0.20	-0.19
^{2}[OC-Fe-CH$_2$OH/ACN]$^{3+}$	0.36	0.52	0.05	0.51	-0.03	-0.44	0.26	-0.13
^{1}[OC-Fe-CH$_2$OH/ACN]$^{2+}$	0.35	0.51	0.14	0.49	-0.07	-0.49	0.19	-0.20
^{1}[OC-Fe-CH$_2$OH]$^{2+}$	0.58	0.48	0.16	0.47	-0.11	-0.47	0.16	-0.19
^{1}[OC-Fe-CH$_2$OH$_2$]$^{3+}$*	0.65	0.47	0.26	0.51	-0.25	-0.27	0.10	-0.18
^{2}[OC-Fe-CH$_2$OH$_2$]$^{2+}$*	0.57	0.47	0.37	0.52	-0.32	-0.30	-0.03	-0.24
^{1}[OC-Fe=CH$_2$]$^{3+}$*	0.62	0.48	0.20	0.50	-0.17	N/A	0.20	-0.12
^{2}[OC-Fe=CH$_2$]$^{2+}$	0.58	0.48	0.27	0.49	-0.55	N/A	0.20	-0.19
^{3}[OC-Fe-CH$_2$-S]$^{3+}$	0.57	0.48	0.19	0.49	-0.31	N/A	0.22	-0.15
^{2}[OC-Fe-CH$_2$-S]$^{2+}$	0.53	0.49	0.30	0.47	-0.34	N/A	0.17	-0.19
^{2}[OC-Fe-CH$_3$]$^{3+}$	0.62	0.50	0.14	0.51	-0.42	N/A	0.25	-0.12
^{1}[OC-Fe-CH$_3$]$^{2+}$	0.59	0.49	0.26	0.50	-0.53	N/A	0.20	-0.20

Table S7 – Electronic, Zero Point (ZPE), and Free Energies (in au) calculated for the optimized geometries of the vanadium complexes involving the binding of 2CO substrates, from C-C bond formation to the release of C_2H_4/C_2H_6.

Intermediate	E	ZPE	G
$^3[Fe\text{-}COCH_2\text{-}S]^{3+}$	-10082.750050	0.097801	-10082.718204
$^2[Fe\text{-}COCH_2\text{-}S]^{2+}$	-10082.876754	0.097488	-10082.841796
$^4[Fe\text{-}COHCH_2\text{-}S]^{3+}$	-10083.337661	0.108652	-10083.292064
$^3[Fe\text{-}COHCH_2\text{-}S]^{2+}$	-10083.458946	0.108563	-10083.412912
$^3[Fe\text{-}CHOHCH_2\text{-}S]^{3+}$	-10083.951356	0.120436	-10083.893291
$^4[Fe\text{-}CHOHCH_2\text{-}S]^{2+}$	-10084.073589	0.119055	-10084.018063
$^4[Fe\text{-}CHOH_2CH_2\text{-}S]^{3+}$	-10084.526161	0.129534	-10084.460363
$^4[Fe\text{-}CHOH_2CH_2\text{-}S]^{3+}$‡	-10084.522861	0.128484	-10084.458007
$^4[Fe\text{-}CHCH_2\text{-}S]^{3+}$	-10008.048468	0.102593	-10008.007992
$^3[Fe\text{-}CHCH_2\text{-}S]^{2+}$	-10008.174826	0.102327	-10008.134540
$^3[Fe\text{-}CH_2CH_2\text{-}S]^{3+}$	-10008.687598	0.116066	-10008.633120
$^4[Fe\text{-}CH_2CH_2\text{-}S]^{2+}$	-10008.820732	0.113422	-10008.770177
$^4[Fe\text{-}CH_2CH_2]^{2+}$	-10008.843792	0.112841	-10008.794086
$^2[Fe\text{-}COCH_2]^{2+}$	-10082.870936	0.096235	-10082.837094
$^4[Fe\text{-}COCH_3]^{3+}$	-10083.381462	0.108967	-10083.333971
$^3[Fe\text{-}COCH_3]^{2+}$	-10083.508681	0.108976	-10083.463387
$^3[Fe\text{-}COHCH_3]^{3+}$	-10083.967949	0.120582	-10083.910636
$^4[Fe\text{-}COHCH_3]^{2+}$	-10084.081480	0.118738	-10084.026863
$^4[Fe\text{-}CHOHCH_3]^{3+}$	-10084.575603	0.130556	-10084.509065
$^3[Fe\text{-}CHOHCH_3]^{2+}$	-10084.702816	0.130618	-10084.635531
$^3[Fe\text{-}CHOH_2CH_3]^{3+}$	-10085.145970	0.141689	-10085.067786
$^2[Fe\text{-}CHOH_2CH_3]^{2+}$	-10085.251856	0.139711	-10085.175406
$^3[Fe\text{-}CHOH_2CH_3]^{3+}$‡	-10085.142048	0.139932	-10085.065699
$^3[Fe\text{-}CH(CH_3)\text{-}S]^{3+}$	-10008.706930	0.116006	-10008.626953
$^4[Fe\text{-}CH(CH_3)\text{-}S]^{2+}$	-10008.829210	0.114982	-10008.776887
$^4[Fe\text{-}CH_2CH_3]^{3+}$	-10009.315689	0.126115	-10009.252539
$^3[Fe\text{-}CH_2CH_3]^{2+}$	-10009.444057	0.126356	-10009.380231
$^4[S\text{-}CH_2CH_3]^{3+}$	-10009.332950	0.127938	-10009.268096
$^3[S\text{-}CH_2CH_3]^{2+}$	-10009.456316	0.126490	-10009.393256

Table S8 – Mulliken spin population analyses of the transition metals and substrate C/O atoms in the optimized geometries of the vanadium complexes involving the binding of 2CO substrates, from C-C bond formation to the release of C_2H_4/C_2H_6.

Intermediate	V	Fe1	Fe2	Fe3	C	C	O
$^3[Fe\text{-}COCH_2\text{-}S]^{3+}$	-0.97	3.50	2.91	-3.43	-0.05	-0.26	-0.17
$^2[Fe\text{-}COCH_2\text{-}S]^{2+}$	-1.27	3.35	2.52	-3.44	-0.04	-0.24	-0.11
$^4[Fe\text{-}COHCH_2\text{-}S]^{3+}$	-0.91	3.57	3.27	-3.43	0.00	-0.12	-0.02
$^3[Fe\text{-}COHCH_2\text{-}S]^{2+}$	-1.20	3.34	3.22	-3.47	0.01	0.23	-0.03
$^3[Fe\text{-}CHOHCH_2\text{-}S]^{3+}$	-1.00	3.49	2.99	-3.45	0.02	-0.37	-0.09
$^4[Fe\text{-}CHOHCH_2\text{-}S]^{2+}$	-1.15	3.39	3.57	-3.45	0.00	0.12	0.03
$^4[Fe\text{-}CHOH_2CH_2\text{-}S]^{3+}$	-1.12	3.46	3.57	-3.45	0.01	0.04	0.01
$^4[Fe\text{-}CHOH_2CH_2\text{-}S]^{3+}$‡	-1.08	3.52	3.48	-3.45	0.01	0.00	0.00
$^4[Fe\text{-}CHCH_2\text{-}S]^{3+}$	-0.73	3.60	3.35	-3.44	0.04	-0.49	N/A
$^3[Fe\text{-}CHCH_2\text{-}S]^{2+}$	-1.13	3.34	3.33	-3.47	0.05	-0.57	N/A

^{3}[Fe-CH$_2$CH$_2$-S]$^{3+}$	-0.93	3.48	2.79	-3.45	0.03	-0.31	N/A
^{4}[Fe-CH$_2$CH$_2$-S]$^{2+}$	-1.22	3.32	3.70	-3.39	0.00	0.14	N/A
^{4}[Fe-CH$_2$CH$_2$]$^{2+}$	-1.07	3.50	3.42	-3.37	-0.07	-0.05	N/A
^{2}[Fe-COCH$_2$]$^{2+}$	-0.97	3.50	1.87	-3.41	-0.11	-0.10	-0.05
^{4}[Fe-COCH$_3$]$^{3+}$	-0.55	3.58	3.14	-3.43	-0.05	-0.21	-0.12
^{3}[Fe-COCH$_3$]$^{2+}$	-1.14	3.55	2.73	-3.41	-0.04	-0.21	-0.09
^{3}[Fe-COHCH$_3$]$^{3+}$	-0.71	3.58	2.22	-3.43	-0.01	-0.18	-0.02
^{3}[Fe-COHCH$_3$]$^{2+}$	0.17	3.50	2.31	-3.18	0.00	-0.23	-0.03
^{4}[Fe-CHOHCH$_3$]$^{3+}$	-0.70	3.60	3.16	-3.42	0.01	-0.33	-0.07
^{3}[Fe-CHOHCH$_3$]$^{2+}$	-1.23	3.52	2.82	-3.41	0.01	-0.29	-0.04
^{3}[Fe-CHOH$_2$CH$_3$]$^{3+}$	-1.00	3.56	2.40	-3.41	0.00	-0.14	0.00
^{2}[Fe-CHOH$_2$CH$_3$]$^{2+}$	-1.14	3.24	2.31	-3.41	0.00	-0.12	0.00
^{3}[Fe-CHOH$_2$CH$_3$]$^{3+}$‡	-0.88	3.57	2.27	-3.41	0.00	-0.14	0.00
^{3}[Fe-CH(CH$_3$)-S]$^{3+}$	-1.08	3.43	2.94	-3.44	0.02	-0.30	N/A
^{4}[Fe-CH(CH$_3$)-S]$^{2+}$	-1.17	3.41	3.41	-3.33	0.01	0.07	N/A
^{4}[Fe-CH$_2$CH$_3$]$^{3+}$	-0.62	3.60	3.04	-3.41	0.01	-0.31	N/A
^{3}[Fe-CH$_2$CH$_3$]$^{2+}$	-1.16	3.52	2.71	-3.41	0.01	-0.27	N/A
^{4}[S-CH$_2$CH$_3$]$^{3+}$	-0.52	3.37	3.36	-3.51	0.00	0.03	N/A
^{3}[S-CH$_2$CH$_3$]$^{2+}$	-1.15	3.27	3.17	-3.47	0.00	-0.02	N/A

Table S9 – Mulliken atomic charge analyses of the transition metals and substrate C/O atoms in the optimized geometries of the vanadium complexes involving the binding of 2CO substrates, from C-C bond formation to the release of C_2H_4/C_2H_6.

Intermediate	V	Fe1	Fe2	Fe3	C	C	O
^{3}[Fe-COCH$_2$-S]$^{3+}$	0.57	0.46	0.33	0.48	0.08	0.23	-0.26
^{2}[Fe-COCH$_2$-S]$^{2+}$	0.50	0.45	0.34	0.49	-0.10	0.27	-0.36
^{4}[Fe-COHCH$_2$-S]$^{3+}$	0.56	0.48	0.33	0.46	-0.10	0.10	-0.31
^{3}[Fe-COHCH$_2$-S]$^{2+}$	0.52	0.46	0.37	0.46	-0.08	0.02	-0.36
^{3}[Fe-CHOHCH$_2$-S]$^{3+}$	0.54	0.49	0.36	0.46	-0.10	0.07	-0.39
^{4}[Fe-CHOHCH$_2$-S]$^{2+}$	0.50	0.46	0.46	0.48	-0.13	-0.03	-0.48
^{4}[Fe-CHOH$_2$CH$_2$-S]$^{3+}$	0.53	0.47	0.46	0.49	-0.16	-0.15	-0.34
^{4}[Fe-CHOH$_2$CH$_2$-S]$^{3+}$‡	0.53	0.46	0.47	0.49	-0.14	-0.14	-0.45
^{4}[Fe-CHCH$_2$-S]$^{3+}$	0.59	0.49	0.49	0.46	-0.09	-0.33	N/A
^{3}[Fe-CHCH$_2$-S]$^{2+}$	0.53	0.46	0.53	0.47	-0.10	-0.39	N/A
^{3}[Fe-CH$_2$CH$_2$-S]$^{3+}$	0.58	0.46	0.40	0.48	-0.12	-0.24	N/A
^{4}[Fe-CH$_2$CH$_2$-S]$^{2+}$	0.57	0.45	0.54	0.45	-0.13	-0.45	N/A
^{4}[Fe-CH$_2$CH$_2$]$^{2+}$	0.60	0.51	0.41	0.50	-0.26	-0.26	N/A
^{2}[Fe-COCH$_2$]$^{2+}$	0.64	0.51	0.28	0.50	-0.31	0.14	-0.33
^{4}[Fe-COCH$_3$]$^{3+}$	0.63	0.52	0.34	0.51	-0.32	0.30	-0.26
^{3}[Fe-COCH$_3$]$^{2+}$	0.62	0.51	0.30	0.51	-0.34	0.31	-0.36
^{3}[Fe-COHCH$_3$]$^{3+}$	0.62	0.53	0.16	0.49	-0.39	0.34	-0.28
^{3}[Fe-COHCH$_3$]$^{2+}$	0.56	0.52	0.22	0.46	-0.39	0.30	-0.31
^{4}[Fe-CHOHCH$_3$]$^{3+}$	0.62	0.54	0.29	0.49	-0.38	0.18	-0.39
^{3}[Fe-CHOHCH$_3$]$^{2+}$	0.61	0.52	0.27	0.49	-0.40	0.19	-0.44
^{3}[Fe-CHOH$_2$CH$_3$]$^{3+}$	0.61	0.53	0.26	0.41	-0.36	-0.03	-0.27

^{2}[Fe-CHOH$_2$CH$_3$]$^{2+}$	0.56	0.51	0.29	0.42	-0.37	-0.03	-0.28
^{3}[Fe-CHOH$_2$CH$_3$]$^{3+}$‡	0.61	0.54	0.26	0.42	-0.34	0.03	-0.45
^{3}[Fe-CH(CH$_3$)-S]$^{3+}$	0.58	0.47	0.36	0.49	-0.40	0.04	N/A
^{4}[Fe-CH(CH$_3$)-S]$^{2+}$	0.54	0.47	0.46	0.45	-0.40	-0.05	N/A
^{4}[Fe-CH$_2$CH$_3$]$^{3+}$	0.62	0.54	0.32	0.52	-0.40	-0.12	N/A
^{3}[Fe-CH$_2$CH$_3$]$^{2+}$	0.61	0.52	0.31	0.52	-0.39	-0.15	N/A
^{4}[S-CH$_2$CH$_3$]$^{3+}$	0.61	0.45	0.45	0.50	-0.38	-0.14	N/A
^{3}[S-CH$_2$CH$_3$]$^{2+}$	0.55	0.50	0.47	0.49	-0.38	-0.12	N/A

Table S10 - Electronic, Zero Point (ZPE), and Free Energies (in au) calculated for the optimized geometries of the intermediates involved in the first few steps of the reduction mechanism after binding CO on the Mo/V center.

Structure	E	ZPE	G
^{4}[Initial/ACN]$^{3+}$	-9187.108807	0.110233	-9187.074863
^{4}[Mo-CO]$^{3+}$	-9167.653749	0.071858	-9167.643573
^{5}[Mo-CO]$^{2+}$	-9167.776620	0.070709	-9167.768476
^{6}[Mo-CO]$^{1+}$	-9167.881040	0.069411	-9167.874586
^{4}[Mo-CHO]$^{4+}$	-9168.049192	0.081807	-9168.028806
^{5}[Mo-CHO]$^{3+}$	-9168.217173	0.081344	-9168.197804
^{4}[Mo-CHO]$^{2+}$	-9168.360704	0.080700	-9168.342161
^{4}[Mo-CHOH]$^{3+}$	-9168.807168	0.093881	-9168.775462
^{5}[V-CO]$^{3+}$	-10043.457530	0.071705	-10043.447582
^{4}[V-CO]$^{2+}$	-10043.601893	0.071090	-10043.592760
^{5}[V-CO]$^{1+}$	-10043.692742	0.069697	-10043.685899
^{5}[V-CHO]$^{4+}$	-10043.850442	0.081109	-10043.831440
^{4}[V-CHO]$^{3+}$	-10044.014625	0.081372	-10043.995147
^{4}[V-CHO]$^{2+}$	-10044.163639	0.080660	-10044.145078
^{5}[V-CHOH]$^{3+}$	-10044.610667	0.093640	-10044.578918

Table S11 – Mulliken spin population analyses for the transition metal and substrate C/O centers of the optimized geometries of the intermediates involved in the first few steps of the reduction mechanism after binding CO on the Mo/V center.

Intermediate	Mo/V	Fe1	Fe2	Fe3	C	O
^{4}[Initial/ACN]$^{3+}$	-0.62	3.38	3.46	-3.44	N/A	N/A
^{4}[Mo-CO]$^{3+}$	-0.56	3.40	3.40	-3.45	0.00	-0.01
^{5}[Mo-CO]$^{2+}$	-0.03	3.45	3.46	-3.42	0.04	0.01
^{6}[Mo-CO]$^{1+}$	0.43	3.55	3.55	-3.47	0.09	0.06
^{4}[Mo-CHO]$^{4+}$	-0.27	3.51	3.51	-3.41	-0.14	-0.20
^{5}[Mo-CHO]$^{3+}$	-0.47	3.45	3.45	-3.44	0.10	0.09
^{4}[Mo-CHO]$^{2+}$	-0.67	3.39	3.39	-3.44	0.03	0.00
^{4}[Mo-CHOH]$^{3+}$	-0.61	3.42	3.38	-3.44	0.01	-0.01
^{5}[V-CO]$^{3+}$	-0.71	3.58	3.57	-3.43	0.02	-0.01
^{4}[V-CO]$^{2+}$	-1.29	3.51	3.51	-3.41	0.01	-0.02
^{5}[V-CO]$^{1+}$	-0.57	3.49	3.49	-3.16	-0.05	-0.03
^{5}[V-CHO]$^{4+}$	-0.16	3.59	3.59	-3.47	-0.14	-0.16
^{4}[V-CHO]$^{3+}$	-0.62	3.48	3.48	-3.42	-0.19	-0.20

^{5}[V-CHO]$^{2+}$	-0.70	3.50	3.50	-3.44	0.14	0.08
^{5}[V-CHOH]$^{3+}$	-0.49	3.54	3.50	-3.46	0.07	0.01

Table S12 – Mulliken atomic charge analyses for the transition metal and substrate C/O centers of the optimized geometries of the intermediates involved in the first few steps of the reduction mechanism after binding CO on the Mo/V center.

Intermediate	Mo/V	Fe1	Fe2	Fe3	C	O
^{4}[InitialMo/ACN]$^{3+}$	0.37	0.55	0.55	0.55	N/A	N/A
^{4}[Mo-CO]$^{3+}$	0.20	0.56	0.56	0.55	0.30	-0.05
^{5}[Mo-CO]$^{2+}$	0.22	0.57	0.57	0.53	0.24	-0.15
^{6}[Mo-CO]$^{1+}$	0.31	0.57	0.57	0.51	0.16	-0.31
^{4}[Mo-CHO]$^{4+}$	0.32	0.56	0.56	0.54	0.17	-0.20
^{5}[Mo-CHO]$^{3+}$	0.34	0.55	0.55	0.54	0.14	-0.30
^{4}[Mo-CHO]$^{2+}$	0.33	0.54	0.54	0.54	0.11	-0.38
^{4}[Mo-CHOH]$^{3+}$	0.29	0.55	0.55	0.55	0.10	-0.28
^{5}[V-CO]$^{3+}$	0.28	0.52	0.52	0.49	0.31	-0.06
^{4}[V-CO]$^{2+}$	0.30	0.50	0.50	0.49	0.30	-0.10
^{5}[V-CO]$^{1+}$	0.24	0.51	0.51	0.45	0.29	-0.16
^{5}[V-CHO]$^{4+}$	0.35	0.53	0.53	0.50	0.19	-0.15
^{4}[V-CHO]$^{3+}$	0.35	0.51	0.51	0.48	0.19	-0.20
^{5}[V-CHO]$^{2+}$	0.36	0.51	0.51	0.48	0.17	-0.34
^{5}[V-CHOH]$^{3+}$	0.35	0.51	0.52	0.47	0.14	-0.31

Table S13 - Electronic, Zero Point (ZPE), and Free Energies (in au) calculated for the optimized geometries of supplementary structures such as by-products and protonation/reducing agents.

Structure	E	ZPE	G
^{1}Acetonitrile	-132.825000	0.044750	-132.804196
^{1}CO	-113.354989	0.004848	-113.369277
^{1}Lutidine	-327.093604	0.142258	-326.982316
^{1}Lutidinium Acid	-327.561570	0.156335	-327.436324
^{2}Cobaltocene	-1770.129002	0.164295	-1769.999727
^{1}Cobaltocene^{+}	-1770.014337	0.169181	-1769.878179
^{1}Water	-76.464223	0.020374	-76.461535
^{1}Methane	-40.537611	0.043932	-40.512054
^{1}Ethylene	-78.628579	0.050308	-78.599155
$^{2}CH_2CH_3$	-79.200511	0.058702	-79.165490
$^{2}CH_3$	-39.859814	0.029464	-39.850149
$^{1}CO_2$	-188.669014	0.011327	188.678396

Table S14 - Electronic, Zero Point (ZPE), and Free Energies (in au) calculated for the optimized geometries of other vanadium complex structures.

Structure	E	ZPE	G
^{3}[Fe-CO_2-Fe/ACN]$^{3+}$	-10251.520289	0.119297	-10251.468501
^{1}[Fe-CO_2/ACN]$^{3+}$	-10251.534584	0.120603	-10251.480296
^{8}[Fe-CO_2/ACN]$^{2+}$	-10251.666174	0.118965	-10251.615844
^{4}[Fe-CH_2OH|Fe-CO]$^{3+}$	-10158.576482	0.112893	-10158.527988
^{5}[Fe-CH_2OH|Fe-CO]$^{2+}$	-10158.729418	0.110731	-10158.683294
^{1}[Fe-COH2CH2]$^{3+}$	-10083.890042	0.107487	-10083.844390
